# CCDC183 is essential for cytoplasmic invagination around the flagellum during spermiogenesis and male fertility

**DOI:** 10.1242/dev.201724

**Published:** 2023-10-30

**Authors:** Keisuke Shimada, Masahito Ikawa

**Affiliations:** ^1^ Department of Experimental Genome Research, Research Institute for Microbial Diseases, Osaka University, Osaka 5650871, Japan; ^2^ Regulation of Host Defense Team, Center for Infectious Disease Education and Research, Osaka University, Osaka 5650871, Japan; ^3^ Laboratory of Reproductive Systems Biology, The Institute of Medical Science, The University of Tokyo, Tokyo 108-8639, Japan

**Keywords:** Spermiogenesis, Male infertility, Cytoplasmic invagination, Sperm centrioles, Flagellar formation, Mouse

## Abstract

Sperm flagellum plays a crucial role in male fertility. Here, we generated *Ccdc183* knockout mice using the CRISPR/Cas9 system to reveal the protein function of the testis-specific protein CCDC183 in spermiogenesis. We demonstrated that the absence of CCDC183 causes male infertility with morphological and motility defects in spermatozoa. Owing to the lack of CCDC183, centrioles after elongation of axonemal microtubules do not connect the cell surface and nucleus during spermiogenesis, which causes subsequent loss of cytoplasmic invagination around the flagellum. As a result, the flagellar compartment does not form properly and cytosol-exposed axonemal microtubules collapse during spermiogenesis. In addition, ectopic localization of accessory structures, such as the fibrous sheath and outer dense fibers, and abnormal head shape as a result of abnormal sculpting by the manchette are observed in *Ccdc183* knockout spermatids. Our results indicate that CCDC183 plays an essential role in cytoplasmic invagination around the flagellum to form functional spermatozoa during spermiogenesis.

## Teaser

**Summary:** CCDC183 is essential for cytoplasmic invagination around the flagellum, enabling proper formation of both axonemal microtubules and accessory structures to produce a functional spermatozoon.

## INTRODUCTION

Fusion between spermatozoa (male gametes) and oocytes (female gametes) is essential for the continuation of mammalian species. Spermatozoon have a flagellum, which is a highly conserved organelle present in most animals (Lindemann and Lesich, 2016). By using the flagellum as a driving force, spermatozoa travel long distances to reach female gametes. Therefore, sperm motility plays an imperative role in male fertility in humans (Larsen et al., 2000). An axoneme, a motility apparatus composed of a ‘9+2’ microtubule arrangement, consisting of nine outer doublets surrounding a pair of single central microtubules, is found throughout the tail except for the endpiece. Axonemal microtubules anchor several macromolecular complexes, such as inner dynein arms, outer dynein arms, radial spokes, and the nexin–dynein regulatory complex (Miyata et al., 2020). Early in sperm flagellum formation, axonemal microtubules begin to elongate from one of the two centrioles at the cell surface (Russell et al., 1990; San Agustin et al., 2015). This elongation of the axoneme causes the spermatid plasma membrane to protrude from the cell (Russell et al., 1990). After the centriole pair contacts the nucleus, the nucleus, centriole pair, and axoneme move to the plasma membrane on the opposite side, which induces cytoplasmic invagination around the flagellum by folding the cell membrane inward (Russell et al., 1990). Although this cytoplasmic invagination in early spermatids has been observed for a long time, it has not been studied in much depth until now.

The sperm flagellum can be divided into three parts: midpiece, principal piece and endpiece (Miyata et al., 2020). Each sperm flagellum segment is classified according to the accessory structures surrounding the microtubules. The midpiece consists of spirally arranged mitochondria and outer dense fibers, whereas the principal piece consists of fibrous sheaths and outer dense fibers. In contrast, the endpiece contains no accessory structures. Mouse spermiogenesis is divided into 16 steps (Russell et al., 1990). According to a previous study, the precursor of the fibrous sheath elongates from the distal end of the principal piece proximally in step 2 spermatids (Irons and Clermont, 1982b). Further, it has been reported that outer dense fibers begin to elongate distally from the connecting piece in step 8 spermatids (Irons and Clermont, 1982a). Both accessory structures elongate along the axonemal microtubules, but little is known about the relationship between the axoneme and both accessory structures during spermiogenesis. Moreover, the molecular mechanisms underlying the formation of both fibrous sheaths and outer dense fibers remain unclear. Considering the fact that disruption of the fibrous sheath structure and/or outer dense fibers are linked to male infertility (Zhao et al., 2018; Xu et al., 2020; Kaneda et al., 2022), it is crucial to understand the molecular mechanisms that govern the formation of these structures. To understand the mechanisms, we generated several gene knockout (KO) mouse lines and found that CCDC183 is essential for flagellar formation.

Coiled-coil domain containing 183 (CCDC183), also known as KIAA1984, is a testis-specific protein (Uhlén et al., 2015) expressed in middle and late round spermatids (Hermann et al., 2018). Multiple studies have reported a relationship between CCDC183 and male infertility (Netherton et al., 2018; Siebert-Kuss et al., 2023), but the functions of CCDC183 have not been described owing to the lack of KO animal models. Here, we generated *Ccdc183* KO mice using the CRISPR/Cas9 system and analyzed the resulting phenotype to reveal the protein function of CCDC183 in spermiogenesis.

## RESULTS

### *Ccdc183*-disrupted male mice are sterile owing to abnormal sperm morphology and motility

**Fig. 1.**
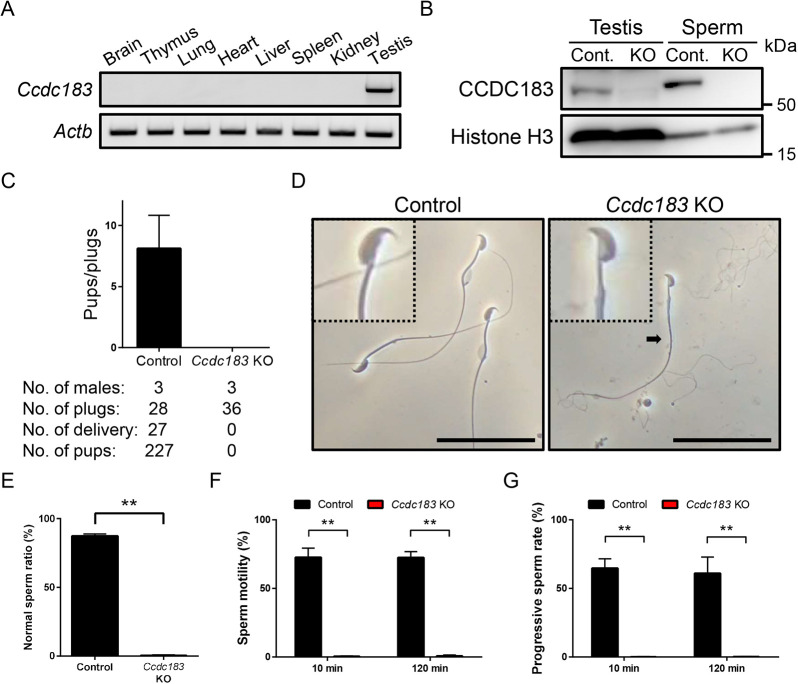
***Ccdc183*-disrupted male mice are sterile with abnormal sperm morphology and motility.** (A) RT-PCR for *Ccdc183* in various mouse tissues. *Actb* was used as a loading control. (B) Protein expression of CCDC183 in testis and cauda epididymal spermatozoa. Histone H3 was used as a loading control. Cont., control. (C) Number of litters born per plug detected. Three males each for control and *Ccdc183* KO were mated with three WT females per male. (D) Observation of spermatozoa obtained from cauda epididymis. Insets show enlarged images of spermatozoa. Although some spermatozoa are relatively normal (arrow), there is a large number of frayed filaments in *Ccdc183* KO cauda epididymis. Scale bars: 50 μm. Images are representative of relatively normal spermatozoa. (E) Proportion of spermatozoa with normal morphology observed by optical microscopy (**P<0.01, two-tailed Student's *t*-test; error bars represent s.d., *n*=3). (F,G) Sperm motility (F) and progressive sperm rate (G) from control and *Ccdc183* KO mice (**P<0.01, two-tailed Student's *t*-test; error bars represent s.d., *n*=3).

To determine the expression profile of *Ccdc183*, we performed RT-PCR using multiple tissues from adult mice. RT-PCR revealed that *Ccdc183* is abundantly expressed in the testis, but not in other tissues (Fig. 1A), consistent with the Human Protein Atlas (Uhlén et al., 2015). As testis-enriched proteins may be involved in spermatogenesis and/or sperm function, we generated *Ccdc183* KO mice using the CRISPR/Cas9 system to reveal the protein function. Guide RNAs targeting areas near the start codon and stop codon were used (Fig. S1A). A mutant line that possessed an 8819 bp deletion in the *Ccdc183* gene was obtained and used for this study (Fig. S1B). To confirm that the CCDC183 protein was lacking in the KO mice, we performed western blot analysis using an anti-CCDC183 antibody. A band near 60 kDa was observed in the control testis and spermatozoa but was absent in the *Ccdc183* KO mice (Fig. 1B), which suggests the KO mice lack CCDC183 protein. *Ccdc183* KO mice are viable and show no overt abnormalities. To test the fertility of the mice, individual male mice [wild type (WT) and homozygous KO] were housed with WT females for 2 months. Although vaginal plugs were observed 36 times, no pups were born from *Ccdc183* KO male mice (Fig. 1C). To reveal the cause(s) of the infertility of the *Ccdc183* KO male mice, we examined spermatozoa obtained from *Ccdc183* KO cauda epididymis. Although a few spermatozoa were relatively normal in *Ccdc183* KO cauda epididymis, a large number of frayed filaments were observed (Fig. 1D). Even spermatozoa that appeared normal had abnormalities when examined closely, such as flagellar thickness (Fig. 1D,E). We then assessed sperm motility using computer-assisted spermatozoa analysis (CASA). CASA revealed that *Ccdc183* KO spermatozoa are incapable of sperm motility and progression (Fig. 1F,G). These results indicate that the causes of infertility in *Ccdc183* KO male mice are abnormal sperm morphology and motility.

### *Ccdc183* KO spermatids exhibit abnormal head shape and flagellar formation

**Fig. 2.**
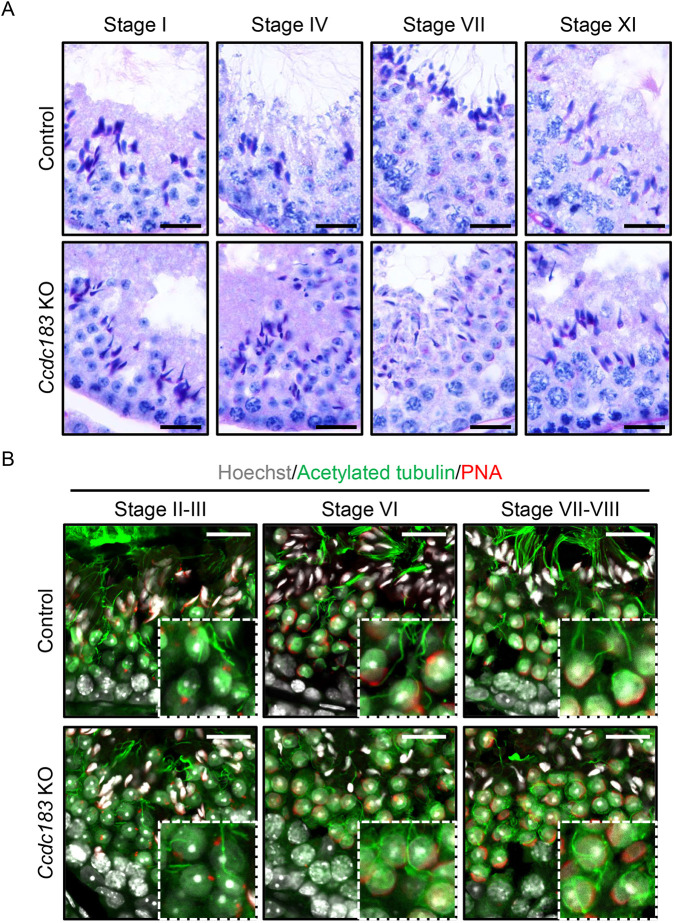
***Ccdc183*-disrupted male mice have fewer sperm flagella in the testis.** (A) PAS staining of testicular sections of adult control and *Ccdc183* KO mice. The sperm flagella in *Ccdc183* KO testis are hard to observe. There are abnormalities in the head shape of elongating spermatids from the *Ccdc183* KO mouse. Scale bars: 20 μm. (B) Immunostaining of microtubules in control and *Ccdc183* KO testis. Testes were stained with acetylated tubulin (green) to visualize microtubules. Hoechst 33342 (white) and PNA-lectin (red) were used to visualize the nuclei and acrosome, respectively. Insets show enlarged images of round spermatids. The number of sperm tails in the lumen of *Ccdc183* KO seminiferous tubules is dramatically lower than control. However, relatively high numbers of axoneme microtubules could be observed around round spermatids. Scale bars: 20 μm.

Our next step was to determine the mechanism of abnormal sperm formation in *Ccdc183* KO mice. The *Ccdc183* KO testes were smaller and lighter than controls (Fig. S2A,B). When we observed histology of testis cross-sections with periodic acid-Schiff (PAS) staining, seminiferous tubules with small lumens were observed (Fig. S2C). In control step 16 spermatids, sperm release to the lumen occurs, and lots of sperm tails are visible in the lumen. However, sperm tails were hard to find in *Ccdc183* KO seminiferous tubules (Fig. S2C). In addition, we had difficulty finding spermatozoa of normal appearance in the *Ccdc183* KO cauda epididymis (Fig. S2D). There were, however, sloughed germ cells observed within the KO epididymis, which may contribute to the reduced testicular weight. To observe sperm formation more clearly, testis sections were observed at high magnification. At all stages, KO sperm tails were difficult to find, and the head shapes of KO spermatids after chromatin condensation were abnormal (Fig. 2A). We also found abnormal residual cytoplasm on the lumen side of KO elongated spermatids (Fig. 2A, Stage VII), which foreshadows abnormal spermiation (Shi et al., 2019; Shimada et al., 2023). As proper manchette formation and removal are crucial for proper sperm head development (Lehti et al., 2013; Lehti and Sironen, 2016), we stained the manchette using anti-α-tubulin antibody and found that *Ccdc183* KO spermatids had abnormalities in manchette localization (Fig. S3A), but not in manchette microtubule structures (Fig. S3B). These results suggest that the abnormal head shape in *Ccdc183* KO spermatozoa is caused by abnormal sculpting by the manchette. In addition, it was difficult to find axonemal microtubules in the KO spermatids, although manchette microtubules were found (Fig. S3A). To check whether sperm flagella were formed in the KO testis, we immunostained the testis against acetylation of α-tubulin (αK40, acetylated tubulin) (LeDizet and Piperno, 1987; Janke and Magiera, 2020). Spermatid flagella were observed in *Ccdc183* KO testis, but most were located around round spermatids and few were found in the lumen of seminiferous tubules (Fig. 2B).

### *Ccdc183* KO spermatids exhibit abnormal cytoplasmic invagination around the flagellum, which causes axonemal microtubule collapse

**Fig. 3.**
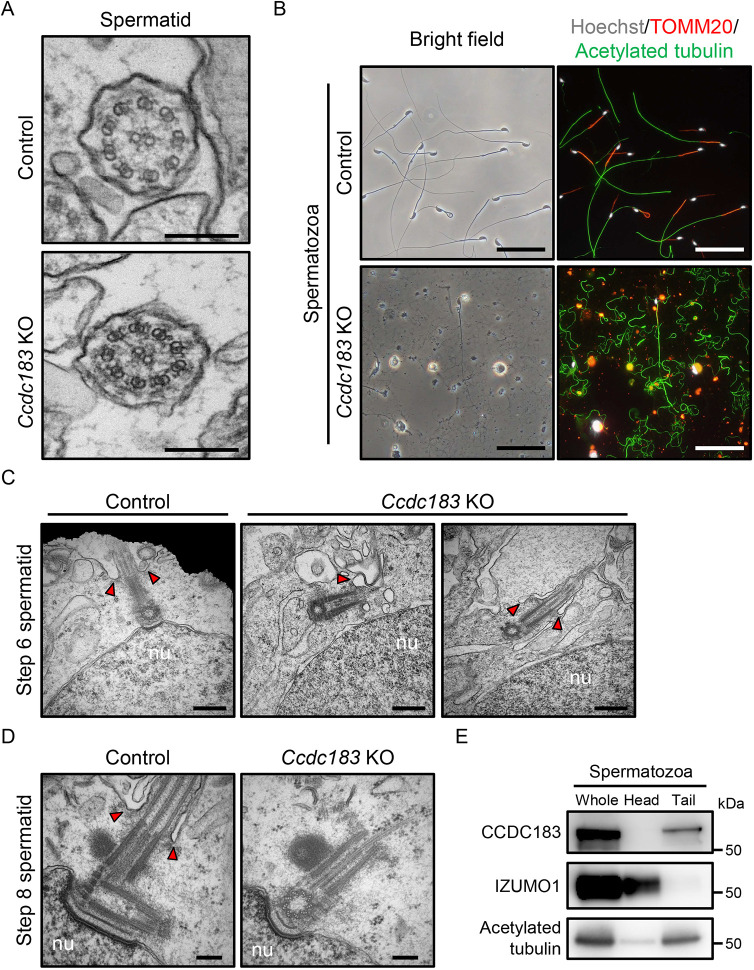
***Ccdc183* KO spermatids have abnormalities in cytoplasmic invagination around the flagellum during spermiogenesis.** (A) Ultrastructural images of the axoneme in step 6 spermatids analyzed by TEM. The ‘9+2’ arrangement of microtubule doublets appears to be normal in *Ccdc183* mutants. Scale bars: 200 nm. *n=*12 spermatids were assessed. (B) Spermatozoa collected from control and *Ccdc183* KO cauda epididymis were stained with TOMM20 (red) and acetylated tubulin (green) to visualize mitochondria and microtubules, respectively. Hoechst 33342 (white) was used to visualize nuclei. The identity of the frayed filaments observed in *Ccdc183* KO epididymis was confirmed to be microtubules. Scale bars: 50 μm. (C) Ultrastructural images of centrioles in step 6 spermatids. Centrioles in control spermatids contact both the cell surface and the nucleus. Centrioles in *Ccdc183* KO spermatid contact the cell surface, but not the nucleus. Arrowheads indicate infolded cell membrane. Scale bars: 500 nm. nu, nuclei. *n=*6 spermatids were assessed. (D) Ultrastructural images of centrioles in step 8 spermatids. A sperm nucleus moves toward the cell surface at the opposite pole, and centrioles follow it, causing the cytoplasm to invaginate inward. Centrioles in *Ccdc183* KO spermatids also follow the sperm nucleus, but no cytoplasmic invagination was observed. Arrowheads indicate cytoplasmic invagination. Scale bars: 200 nm. nu, nuclei. *n=*5 spermatids were assessed. (E) Western blot analysis using proteins collected from the sperm head and tail. CCDC183 was detected in the tail fraction. IZUMO1 and acetylated tubulin were detected as markers for heads and tails, respectively.

We performed ultrastructural analysis using transmission electron microscopy (TEM) to analyze the sperm flagella in more detail. We could observe normal ‘9+2’ axonemal microtubule structures in the flagellum in KO spermatids (Fig. 3A). However, we were unable to find normal ‘9+2’ microtubule structures with accessory structures during spermiogenesis (Fig. S4A,B). Instead, we found mitochondrial sheaths without normal axoneme structure in step 16 KO spermatids (Fig. S4A,B). Examination of *Ccdc183* KO spermatozoa collected from the cauda epididymis revealed that spermatozoa had incomplete membranes and substances with high electron density (Fig. S4C), which foreshadows cell death (Shimada et al., 2023). We could not find normal axoneme structures in spermatozoa, but fragmented microtubules attached to outer dense fibers were found (Fig. S4D). We then immunostained the spermatozoa with acetylated tubulin and found that microtubules were scattered in the KO cauda epididymis (Fig. 3B). These results suggest that, although *Ccdc183* KO spermatids could form normal axonemal structures, the ‘9+2’ microtubule structures in *Ccdc183* KO spermatozoa collapse during spermiogenesis.

To observe axonemal microtubule behavior in more detail, we immunolabeled acetylated tubulin in KO testis and analyzed them using TEM. Most microtubule bundles in *Ccdc183* KO spermatids were located within the cytoplasm without a flagellar structure (Fig. S5A, arrows). In addition, broken microtubule bundles were observed in *Ccdc183* KO spermatids (Fig. S5A, arrowheads). These results suggest that *Ccdc183* KO spermatids have a problem with axonemal microtubule stability. However, microtubules with intact axonemal structures were also observed in the *Ccdc183* KO testicular lumen, as in Fig. 3A (Fig. S5B). These results suggest that axonemes within the flagellum have microtubule stability, but not axonemes within the cytoplasm. Next, we focused on centrioles, which nucleate axonemal microtubule elongation (Russell et al., 1990). The centriole pair connects the cell surface and nucleus in step 6 spermatids, but this could not be observed in *Ccdc183* KO spermatids (Fig. 3C). After step 6, the sperm nucleus, centriole pairs, and axoneme move toward the cell surface on opposite sides, which allows cytoplasmic invagination around the flagellum (Russell et al., 1990). However, no cytoplasmic invagination was observed around the axoneme in step 8 spermatids, although the centrioles migrated with the nucleus (Fig. 3D). These results suggest that CCDC183 is essential for proper cytoplasmic invagination around the flagellum during spermiogenesis.

To check the localization of CCDC183, we tried immunofluorescence analysis using an anti-CCDC183 antibody, but the antibody did not work. Therefore, we separated sperm heads and tails for immunoblotting analysis and detected the CCDC183 band in the tail fraction (Fig. 3E). In addition, we fractionated sperm protein using Triton X-100 and sodium dodecyl sulfate (SDS). CCDC183 protein was mainly present in the SDS-soluble fraction (Fig. S5C), which indicates that CCDC183 may be associated with the axonemal microtubules (Carrera et al., 1994). In addition, some CCDC183 remained in the SDS-resistant fraction, which may be associated with the accessory structures.

### *Ccdc183* KO spermatids have abnormalities in accessory structure localization

**Fig. 4.**
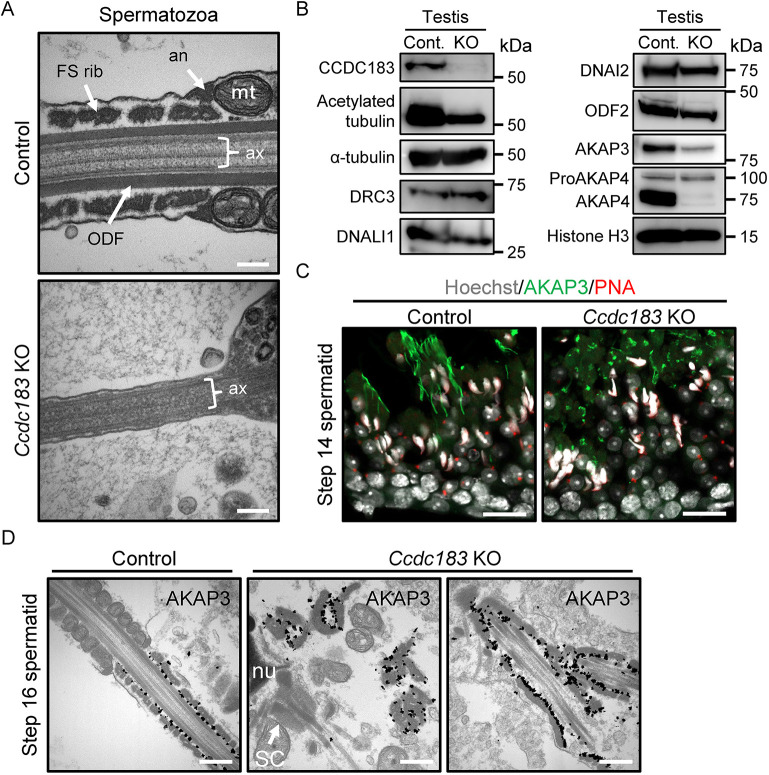
***Ccdc183*-disrupted male mice show abnormal fibrous sheath localization.** (A) Longitudinal sections of sperm tails in the cauda epididymis observed by TEM. *Ccdc183* KO sperm tails contain dense materials inside the flagellum without a fibrous sheath or outer dense fiber structures. A faint axoneme is observed within the KO sperm flagellum. Scale bars: 200 nm. an, annulus; ax, axoneme; FS rib, fibrous sheath rib; mt, mitochondrion; ODF, outer dense fiber. *n=*12 spermatozoa were assessed. (B) Protein expression related to microtubules, outer dense fibers, and fibrous sheath in control and *Ccdc183* KO testis. Histone H3 was used as a loading control. Cont., control. (C) Immunostaining of the fibrous sheath in control and *Ccdc183* KO testis. Testes were stained with AKAP3 (green) to visualize the fibrous sheath. Hoechst 33342 (white) and PNA-lectin (red) were used to visualize the nuclei and acrosome, respectively. Spermatids in *Ccdc183* KO fail to elongate their fibrous sheath. Scale bars: 20 μm. (D) Detection of immunolabeled AKAP3 in step 16 spermatid observed by TEM using anti-AKAP3 antibody incubated with 1.4-nm gold particle-conjugated secondary antibody. In the control spermatid, AKAP3 was detected on the inner side of the fibrous sheath surface. In *Ccdc183* KO spermatids, fibrous sheaths could be observed scattered in the cytoplasm when AKAP3 was used as an indicator (center panel). Although it was very rare, we also observed a fibrous sheath surrounding axonal microtubules and outer dense fibers (right panel). Scale bars: 500 nm. nu, nuclei; SC, segmented column.

When we examined the principal piece in *Ccdc183* KO spermatozoa using TEM, almost all principal pieces did not have fibrous sheath and outer dense fibers, although a faint axoneme was observed (Fig. 4A). Therefore, we checked the amount of proteins related to axonemal microtubules and accessory structures in *Ccdc183* KO testis. Although the expression level of acetylated tubulin (stable microtubules) was decreased, those of proteins contained in the axoneme, such as α-tubulin (microtubule), DRC3 (dynein regulatory complex), DNALI1 (inner dynein arm) and DNAI2 (outer dynein arm), were not changed in *Ccdc183* KO testis (Fig. 4B). Based on these results, we conclude that the amount of protein forming axonemal microtubules itself has not changed, but the amount of stable microtubules (Li and Yang, 2015) has decreased, which reflects abnormal axonemal formation in *Ccdc183* KO testis. We also found that the expression signals of the fibrous sheath-associated proteins AKAP3 and AKAP4 were dramatically decreased, but not that of outer dense fiber-associated protein [ODF2; also known as ODF84 (Shao et al., 1997)] or the precursor of AKAP4 (proAKAP4) (Fig. 4B). Therefore, we next focused on the fibrous sheath. A previous study revealed AKAP3 is detected from step 4 spermatids to mature spermatozoa and AKAP4 from step 14 spermatids to mature spermatozoa (Brown et al., 2003). Therefore, we examined the localization of AKAP3 in late spermiogenesis. AKAP3 was detected in filament-like structures in control, but as dots without filament-like signals in *Ccdc183* KO elongated spermatids (Fig. 4C). Because the fibrous sheath extends from the distal end of the principal piece to the annulus (proximal end) along the axonemal microtubules (Irons and Clermont, 1982b), it appears that little elongation of the fibrous sheath occurred in *Ccdc183* KO testis. Examination of AKAP3 by immuno-electron microscopy revealed that AKAP3 was observed scattered in the cytoplasm (Fig. 4D, center). Although it was very rare, we also observed a fibrous sheath surrounding axonemal microtubules and outer dense fibers (Fig. 4D, right). However, these structures were not compartmentalized by a flagellar membrane (Fig. 4D, right). These results indicate that the fibrous sheath localizes at the correct location if axonemal microtubules are correctly present, but it is difficult for fibrous sheath-component proteins to enter the flagellar compartment.

**Fig. 5.**
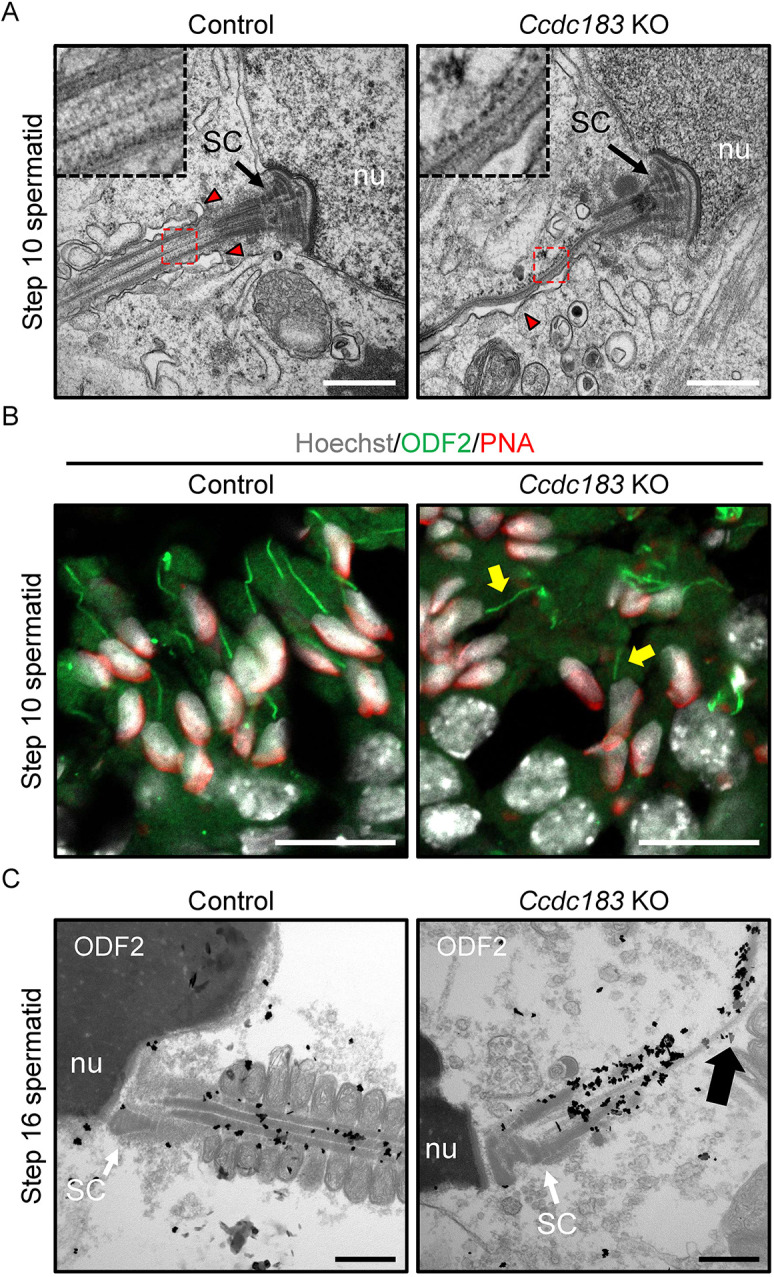
***Ccdc183*-disrupted male mice have abnormal outer dense fiber localization.** (A) Longitudinal sections of the connecting piece in step 10 spermatids observed by TEM. Insets show enlarged images of the boxed area. Outer dense fibers are not correctly localized at the proximal end of the sperm tail where outer dense fibers should start to elongate toward the distal end. It is noted that abnormalities exist in both axonemal microtubule structures and cytoplasmic invagination in *Ccdc183* KO spermatids. Arrowheads indicate cytoplasmic invagination. Scale bars: 1 μm. nu, nuclei; SC, segmented column. *n=*3 spermatids were assessed. (B) Immunostaining of outer dense fibers in control and *Ccdc183* KO testis. Testes were stained with ODF2 (green) to visualize outer dense fibers. Hoechst 33342 (white) and PNA-lectin (red) were used to visualize the nuclei and acrosome, respectively. Several ODF2 proteins were localized correctly (arrows) in *Ccdc183* KO spermatids, but most localized ectopically. Scale bars: 20 μm. (C) Detection of immunolabeled ODF2 in step 16 spermatid observed by TEM using an anti-ODF2 antibody incubated with 1.4-nm gold particle-conjugated secondary antibody. Although the correct outer dense fiber structure was rarely observed in *Ccdc183* KO spermatids, ODF2 could localize around the axonemal microtubules. Arrow indicates a broken axonemal microtubule. Scale bars: 500 nm. nu, nuclei; SC, segmented column.

As we observed the absence of outer dense fibers in the KO principal piece (Fig. 4A), we studied the period shortly after the formation of outer dense fibers in step 8 spermatids (Irons and Clermont, 1982a). Although the connecting piece was normal, the structures of the axonemal microtubules and outer dense fibers were abnormal (Fig. 5A). Furthermore, *Ccdc183* KO spermatids did not exhibit correct cytoplasmic invagination around the flagellum (Fig. 5A, right, arrowhead). Immunofluorescence analysis revealed that most ODF2 was ectopically localized in step 10 spermatids, but some ODF2 localized normally (Fig. 5B). Based on immunoelectron microscopy, ODF2 could localize around the axonemal microtubules, despite the absence of normal axoneme and outer dense fiber structures (Fig. 5C).

## DISCUSSION

**Fig. 6.**
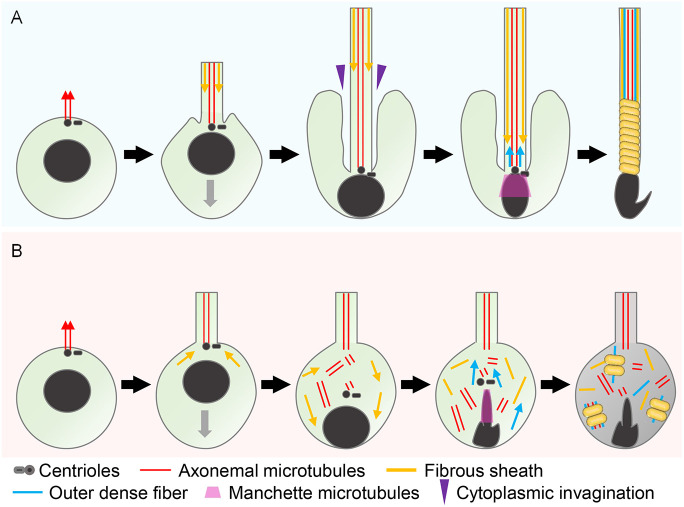
**Schematic model of abnormal sperm formation in *Ccdc183* KO spermatids.** (A) Schematic model of flagellum formation in WT spermatids. Axonemal microtubules elongate from centrioles localized on the cell surface (left-most panel). The spermatid nucleus contacts the centrioles, and the fibrous sheath columns form at the distal end in a distal-to-proximal direction (second from the left). The spermatid nucleus and centrioles move toward the cell surface on opposite sides, which causes cytoplasmic invagination around the flagellum (center). While outer dense fibers form in a proximal-to-distal direction, the manchette is formed around the posterior side of the nucleus to sculpt the nucleus (second from the right). A spermatozoon with accessory structures (outer dense fibers and fibrous sheath) is formed (right-most panel). (B) Schematic model of flagellum formation in *Ccdc183* KO spermatids. *Ccdc183* KO spermatids have abnormalities in the connection between centrioles and the nucleus (second from the left), which cause cytoplasmic invagination failure (center). Owing to the cytoplasmic invagination failure, axonemal microtubules within the cytoplasm collapse during spermiogenesis, and exhibit subsequent abnormal localization of accessory structures. In addition, abnormal head shapes form as a result of abnormal manchette sculpting (second from the right). The *Ccdc183* KO spermatozoon has a huge cytoplasmic area with abundant cytoplasmic contents including electron-dense materials (right). Note that fibrous sheath-component proteins cannot enter the flagellar compartment during spermiogenesis in *Ccdc183* KO spermatids.

In the present study, we demonstrated that absence of CCDC183 induces male infertility with morphological and motility defects in spermatozoa. Examination of sperm morphology in the *Ccdc183* KO epididymis revealed no normal-shaped spermatozoa (Fig. 1D,E). Instead, we found many scattered microtubules in the KO epididymis (Fig. 3B). Therefore, we originally thought that *Ccdc183* KO male mice would have abnormalities in the formation of sperm axonemes, but we could observe normal axonemal structures within the flagellum (Fig. 3A, Fig. S5B). There was, however, disintegration of the axonemal structures within the cytoplasm (Fig. S5A). These results suggest that the axonemal structures of *Ccdc183* KO spermatids collapse during spermiogenesis. In addition, the collapse of axonemal microtubules was also observed by α-tubulin staining of spermatids (Fig. S3A). Because axonemal structures within the flagellum (compartmentalized axonemes) are intact, but those left in the cytoplasm (cytosol-exposed axonemes) are collapsed, there seem to be substantial differences between compartmentalized and cytosol-exposed axonemal microtubules. Axonemal microtubules begin to elongate from one of the two centrioles at the cell surface during steps 2-3 (Fig. 6A, leftmost) (Russell et al., 1990; San Agustin et al., 2015). At this time, the axoneme causes the spermatid plasma membrane to protrude from the cell (Fig. 6A, second from the left) (Russell et al., 1990). The axoneme is tightly surrounded by the flagellar membrane, which continues with the cellular plasma membrane (San Agustin et al., 2015). The centriole pair with the axoneme contacts the nucleus, and the plasma membrane attached to the centriole folds inward (Russell et al., 1990). Then, the sperm nucleus moves toward the cell surface on opposite sides (Russell et al., 1990). Centrioles and the flagellum follow the sperm nucleus, causing cytoplasmic invagination around the flagellum (Fig. 6A, center). As a result, cytosol-exposed axonemes are not detectable during normal spermiogenesis in mammals. *Ccdc183* KO spermiogenesis (Fig. 6B) shows a failure in cytoplasmic invagination around the flagellum (Figs 3D, 5A), which causes abnormal cytosol-exposed axonemes (Fig. S5A). Consequently, cytosol-exposed axonemal microtubules collapse during spermiogenesis (Fig. 6B, center) owing to the absence of accessory structures around the axoneme, protection by the flagellum, or other factors, but compartmentalized axonemal microtubules do not collapse (Fig. 3A, Fig. S5B). Thus, elongated spermatids with flagellar compartmentalization failure are formed (Fig. 6B, rightmost). In contrast to mammals, *Drosophila* spermatids have compartmentalized axonemes in a limited area, and most axonemes are cytosol-exposed axonemes (Tokuyasu, 1975; Avidor-Reiss and Leroux, 2015). As an ortholog of mouse CCDC183 (or KIAA1984) could not be identified in *Drosophila* by InParanoid 8 analysis (Sonnhammer and Östlund, 2015), CCDC183 may be an evolutionarily essential component of axoneme compartmentalization in mammals.

During steps 2-3, the precursors or anlagen of the fibrous sheath columns begin to form at the distal end of the principal piece and gradually extend in a proximal direction (Irons and Clermont, 1982b). The anlagen of the longitudinal columns are joined to the outer aspects of microtubule doublets 3 and 8, and elongate proximally along the axoneme (Irons and Clermont, 1982b). Subsequently, the fibrous sheath columns and ribs are assembled in a distal-to-proximal direction in what will become the principal piece of the spermatozoa (Irons and Clermont, 1982b). By contrast, outer dense fibers appear along the most proximal portion of the axoneme in step 8 spermatids (Irons and Clermont, 1982a). Subsequently, outer dense fibers accumulate in thick layers in a proximal-to-distal direction extending throughout the midpiece and principal piece (Oko, 1998). Through these processes, the sperm flagella and accessory structures are properly formed (Fig. 6A, rightmost). However, the fibrous sheath and its component protein could not be detected in *Ccdc183* KO flagellum (Fig. 4A). Failure of flagellar compartmentalization due to failure of cytoplasmic invagination seems to have resulted in failure of fibrous sheath-component protein entry into the flagellum compartment and/or a defect in protein delivery within the flagellum compartment via intra-flagellar transport (IFT) (Pleuger et al., 2020). Considering that mislocalization of fibrous sheath and outer dense fibers as a result of mutation of IFT component genes has been observed in several KO mouse models (Zhang et al., 2016; Liu et al., 2017; Zhang et al., 2017, 2018; Shi et al., 2019), similar events might have happened in *Ccdc183* KO spermatids as well.

*Ccdc183* KO spermatids have abnormalities in the localization of the outer dense fibers (Fig. 5A,B). Given that outer dense fiber-component proteins localized correctly (Fig. 5C), ectopic localization of outer dense fibers is likely caused by the collapse of axonemal microtubule structures. The abnormal sperm head shape observed in *Ccdc183* KO spermatids (Fig. 2A, Fig. S3A) may also be caused by the collapse of the axoneme. This means a head shape defect caused by abnormal manchette sculpting (Fig. S3A) is thought to be an indirect phenotype of CCDC183 disruption because ectopic localization of centrioles (step 6) was observed before manchette formation (step 8). Although it is under debate, manchette microtubules might be nucleated from the centrosome (Lehti and Sironen, 2016). Therefore, it might be a reasonable assumption that ectopic centriole localization and axoneme collapse affect manchette formation in *Ccdc183* KO spermatids.

A previous study revealed that CCDC183 is highly abundant in good-quality human spermatozoa, but scarce in poor-quality spermatozoa (Netherton et al., 2018). As we have demonstrated that the absence of CCDC183 causes abnormal sperm morphology and motility, our conclusions are consistent with the previous study. The same paper also mentioned that ‘the ortholog of CCDC183 in *Drosophila* (*CCDC151*) is essential for the proper construction of cilia’ (Netherton et al., 2018). It is true that mutations in *CCDC151* result in the absence of both inner and outer dynein arms in respiratory cilia of *Drosophila* (Alsaadi et al., 2014). However, there is no evidence that *Drosophila CCDC151* is an ortholog of mouse CCDC183. As mentioned above, there is no ortholog of mouse CCDC183 in *Drosophila*, and the ortholog of *Drosophila CCDC151* is mouse ODAD3 (also known as CCDC151). Because disruption of ODAD3 causes a lack of outer dynein arms in humans (Hjeij et al., 2014), mammalian ODAD3 (CCDC151) seems to be an ortholog of *Drosophila CCDC151.* Furthermore, we observed both inner and outer dynein arms in *Ccdc183* KO spermatids (Fig. 3A), unlike in *CCDC151*-disrupted *Drosophila* (Alsaadi et al., 2014). Thus, mouse CCDC183 may have a different ortholog than *Drosophila CCDC151*.

In summary, our results suggest that CCDC183 is essential for male fertility as a result of its role in cytoplasmic invagination around the flagellum during spermiogenesis. CCDC183 is essential for appropriate centriole behavior in early spermatids, allowing correct cytoplasmic invagination around the flagellum, which enables a proper flagellar compartment. However, because of the inability to determine the subcellular localization of CCDC183 and the proteins that interact with CCDC183 owing to the lack of suitable antibodies, the molecular mechanisms underlying CCDC183 function still remain unclear. Although further studies are necessary, this study contributes to our understanding of flagellar biogenesis during spermatogenesis, which could aid the diagnosis of male infertility and improve male infertility treatments in the future.

## MATERIALS AND METHODS

### Animals

All animal experiments were approved by the Animal Care and Use committee of the Research Institute for Microbial Diseases, Osaka University. Animals were housed in a temperature-controlled environment with 12 h light cycles and free access to food and water. B6D2F1 (C57BL/6×DBA2), ICR or C57BL6/J mice were used as embryo donors, as foster mothers or for RNA extraction, respectively. These animals were purchased from CLEA Japan, Inc. (Tokyo, Japan) or Japan SLC (Shizuoka, Japan).

### Isolation of RNA and RT-PCR

Isolation of RNA and RT-PCR was performed as previously described (Morohoshi et al., 2020). RNA was isolated and purified from multiple adult tissues of C57BL/6N mice with TRIzol (Thermo Fisher Scientific). Reverse transcription was performed using SuperScript IV Reverse Transcriptase (Thermo Fisher Scientific) with an oligo (dT) primer. PCR was carried out using KOD FX Neo (TOYOBO). The primers used in this study are listed in Table S1.

### Generation of *Ccdc183* KO mice

*Ccdc183* KO mice were generated as previously described (Abbasi et al., 2018). We designed two gRNAs to recognize exon 1 and exon 14 to remove the whole coding sequence (Fig. S1A). The crRNA sequences used in this study were 5′-AGGCACAGATAACGGAGCTA-3′ and 5′- ACCATAGTTACGTCCCTTCG-3′. Synthesized crRNAs (Merck), tracrRNA (Merck) and CAS9 protein (Thermo Fisher Scientific) were incubated to make the CAS9 ribonucleoprotein (RNP) complex. The obtained complex was electroporated into fertilized eggs using a NEPA21 electroporator (Nepa Gene). Of the 120 fertilized eggs that had been electroporated, 112 eggs were transplanted into the oviducts of pseudopregnant females. A total of 32 potential founder mice (F0) were born, and 18 pups possessed mutations. *Ccdc183* KO mice were maintained by sibling crosses. Male mice over 12 weeks of age were used for the studies.

Frozen spermatozoa from *Ccdc183* KO males (B6D2-*Ccdc183^em1Osb^*) were deposited at both the Riken BioResource Center, Ibaraki, Japan (RBRC number: 11642) and the Center for Animal Resources and Development (CARD), Kumamoto University, Kumamoto, Japan (CARD ID: 3199). *Ccdc183* KO mice are available through these centers.

### Genotyping analysis

PCR was performed using KOD FX Neo. The primers used in this study are listed in Table S1.

### Generation of antibodies

A rabbit polyclonal antibody was produced by immunization with mouse CCDC183 polypeptide (C plus IDKIHTKETSEKYRRGR). The CCDC183 antibody was purified from serum using the CCDC183 polypeptide and SulfoLink coupling resin (Thermo Fisher Scientific). Antibodies against SLC2A3 (KS64-10), IZUMO1 (KS064-125) and RSPH6A used in this study were generated as previously described (Fujihara et al., 2013; Inoue et al., 2013; Abbasi et al., 2018).

### Immunoblot analysis

Immunoblot analysis was conducted as previously described (Shimada et al., 2021). For extracting whole proteins from testis or spermatozoa, urea lysis buffer (6 M urea, 2 M thiourea and 2% sodium deoxycholate) was used. Samples were subjected to SDS-PAGE followed by western blotting. After blocking with 10% skim milk, blots were incubated with primary antibodies overnight at 4°C and then incubated with secondary antibodies conjugated to horseradish peroxidase for 2 h at room temperature. The antibodies used in this study and their dilution conditions are listed in Table S2.

### *In vivo* male fertility test

To confirm the fertility of *Ccdc183* KO male mice, natural mating tests were conducted. Three male mice were individually caged with three B6D2F1 females for 2 months. Both plug and pup numbers were checked at approximately 10:00 h every weekday to determine the number of copulations and litter size.

### Morphological and histological analysis

Spermatozoa were collected from the cauda epididymis and suspended in TYH medium (Toyoda and Yokoyama, 2016). A sperm suspension was mounted on a MAS-coated glass slide (Matsunami Glass, Osaka, Japan), and a cover slip (Matsunami) was added. Sperm morphology was observed using a BX53 microscope (Olympus).

Morphological and histological analysis of testis was conducted as previously described (Shimada et al., 2019). Male mice were euthanized and testes were dissected. After measuring the testicular weight, testes and epididymides were fixed with Bouin's fixative (Polysciences). Fixed testes and epididymides were embedded in paraffin, sectioned, rehydrated and treated with 1% periodic acid for 10 min, followed by treatment with Schiff's reagent (Wako) for 20 min. The sections were stained with Mayer's Hematoxylin solution prior to imaging, and observed using a BX53 microscope (Olympus).

### Sperm motility analysis

Sperm motility analysis was conducted as described previously (Miyata et al., 2021). Cauda epididymal spermatozoa were suspended and incubated in TYH medium, which can induce sperm capacitation (Toyoda and Yokoyama, 2016). Sperm motility was then measured using the CEROS II sperm analysis system (software version 1.5; Hamilton Thorne Biosciences). The motility of epididymal spermatozoa was recorded after 10 min and 2 h of incubation in TYH medium.

### Manchette staining

Manchette staining was performed as described previously (Abbasi et al., 2018). Germ cells including spermatids were squeezed out from the seminiferous tubules onto slide glasses and air-dried at 37°C. The samples were fixed with 4% paraformaldehyde (PFA) in PBS for 15 min and washed with PBS three times for 5 min each wash. The samples were then permeabilized with 0.1% Triton X-100 for 15 min, washed with PBS three times for 5 min each wash, and blocked with 3% bovine serum albumin (BSA) diluted in PBS for 1 h at room temperature. Then, the samples were incubated overnight at 4°C with primary antibodies. After three washes, the appropriate Alexa Fluor-conjugated secondary antibodies (Thermo Fisher Scientific) were added to the slides and incubated. The samples were stained with Hoechst 33342 (Thermo Fisher Scientific) for visualization of nuclei and coverslipped with Immu-Mount (Thermo Fisher Scientific). The antibodies used in this study are listed in Table S2.

### Immunofluorescence of testes and spermatozoa

Immunofluorescence analysis of testes was performed using cryosections as previously described (Isotani et al., 2005). Testes were fixed with 4% PFA, embedded in OCT compound (Sakura Finetek), and 10 μm sections were prepared with a cryostat (CryoStar NX70, Thermo Fisher Scientific). The sections were subjected to antigen retrieval, permeabilization and blocking. Then, the sections were incubated overnight at 4°C with primary antibodies. After three washes, the appropriate Alexa Fluor-conjugated secondary antibodies and Alexa Fluor-conjugated lectin PNA (Thermo Fisher Scientific) were added to the slides and incubated. The sections were stained with Hoechst 33342 for visualizing nuclei and coverslipped with Immu-Mount.

Immunofluorescence analysis of spermatozoa was performed as previously described (Shimada et al., 2021). Spermatozoa were suspended in PBS, smeared on microscope slides, dried at 37°C for 15 min, fixed with 4% PFA, blocked with 3% BSA for 1 h and immunostained with primary antibodies. Goat anti-rabbit or anti-mouse Alexa Fluor-conjugated secondary antibodies were used as secondary antibodies. The samples were then stained with Hoechst 33342 for visualizing nuclei, and coverslipped.

Microscopic images were obtained using a Nikon Eclipse Ti microscope connected to a C2 confocal module (Nikon). Fluorescent images were false-colored and cropped using ImageJ software (version 2.0.0, NIH, Bethesda, MD, USA). The antibodies used in this study are listed in Table S2.

### Ultrastructural analysis using TEM

Ultrastructural analysis using TEM was conducted as previously described (Shimada et al., 2019). Testes were dissected after perfusion fixation with 4% PFA in PBS under anesthesia, and immersed in 4% PFA for 6 h at 4°C. The organs were sliced into 2 mm thick sections with safety razors, immersed in 1% glutaraldehyde in 30 mM HEPES (pH 7.8) overnight at 4°C, and washed three times (5 min each) in 30 mM HEPES. Tissues were postfixed in 1% OsO_4_ and 0.5% potassium ferrocyanide in 30 mM HEPES for 1 h at room temperature. After being washed with distilled water, samples were dehydrated with a graded series of ethanol solutions at room temperature. Dehydrated samples were incubated twice for 5 min in 100% propylene oxide (PO), and then placed in a mixture of PO and epoxy resin for 1 h at room temperature. Sample tissues were incubated in a pure epoxy resin mixture twice for 1 h at room temperature, and embedded in epoxy resin for 2 days at 60°C. Ultrathin sections (80 nm) were cut and stained with 2% uranyl acetate solution for 30 min, stained with a lead staining solution for 2 min, and washed three times with distilled water. The samples were examined using a JEM-1400 Plus electron microscope (JEOL) at 80 kV with a CCD Veleta 2 K×2 K camera (Olympus). Stages of the epithelial cycle were identified based on the morphological characteristics of the spermatids, in particular their nucleus and acrosomal system (Russell et al., 1990). We evaluated spermatid distinction in steps 1-8 by the elongation of the acrosomal system, steps 9-11 by nuclear morphology, step 12 by the presence of meiotic cells, and steps 13-16 by the round spermatids in the same seminiferous tubules.

Immunoelectron microscopy analysis was performed as previously described (Shimada et al., 2021). Testes were dissected after perfusion fixation with 4% PFA in PBS under anesthesia and sliced into 2 mm thick sections. Sectioned samples were fixed with 4% formaldehyde in 0.1 M phosphate buffer (pH 7.4) and washed with 0.1 M phosphate buffer containing 4% sucrose. For cryo-protection, tissue slices were sequentially incubated in 10%, 15% and 20% sucrose in 0.1 M phosphate buffer, embedded in OCT compound (Sakura), and frozen in liquid nitrogen. Six-micrometer-thick sections were cut at −20°C using a cryostat (CryoStar NX70, Thermo Fisher Scientific), and the cryo-sections were attached to MAS-coated glass coverslips and air-dried. The samples were blocked with blocking solution (0.1 M phosphate buffer containing 0.1% saponin, 10% BSA, 10% normal goat serum and 0.1% cold water fish skin gelatin) for 30 min. The blocking solution was replaced with primary antibody in blocking solution and samples were incubated overnight at 4°C. The sections were washed with 0.1 M phosphate buffer containing 0.005% saponin. Samples were incubated with goat anti-mouse or anti-rabbit IgG coupled to 1.4 nm gold 1:300 (Nanogold, Nanoprobes) in blocking solution as a secondary antibody for 3 h. The samples were washed with 0.1 M phosphate buffer containing 0.005% saponin, followed by washing in 0.1 M phosphate buffer, and then fixed with 1% glutaraldehyde in 0.1 M phosphate buffer for 10 min. The sections were washed in PBS containing 50 mM glycine, followed by washing in PBS containing 1% BSA in water. Gold labeling was intensified with GoldEnhance EM kit (Nanoprobes) for 3 min. The gold intensification solution was removed, the sections were soaked in 1% sodium thiosulfate solution for a few seconds, and washed in water. The sections were post-fixed in 1% OsO_4_ and 1.5% potassium ferrocyanide in 0.1 M phosphate buffer for 1 h. Samples were dehydrated in a graded series of ethanol, substituted with PO, and embedded in epoxy resin. Ultrathin sections (80 nm) were stained with 8% uranyl acetate and lead staining solution. The samples were examined using the same electron microscope as above. The antibodies used in this study are listed in Table S2.

### Fractionation of sperm protein

Sperm head-tail separation was performed as previously described (Kaneda et al., 2023). Spermatozoa obtained from the cauda epididymis were suspended in PBS and sonicated to separate tails from heads on ice (Sonifier SLPe, Branson Ultrasonics). The sample was centrifuged (10,000 ***g*** for 5 min), and the pellet was resuspended in 90% Percoll solution (GE Healthcare) in PBS. After further centrifugation (15,000 ***g*** for 15 min), the sperm heads were at the bottom of the tube, and the sperm tails were at the top. The pellets were dissolved in a lysis buffer containing 6 M urea, 2 M thiourea and 2% sodium deoxycholate.

Sperm protein fractionation was performed as described previously (Cao et al., 2006; Castaneda et al., 2017) with slight modification. Spermatozoa were suspended in 1% Triton X-100 lysis buffer (50 mM NaCl, 20 mM Tris-HCl, pH 7.5, protease inhibitor mixture) and incubated for 2 h at 4°C. The sample was centrifuged at 15,000 ***g*** for 10 min to separate the Triton-soluble fraction (supernatant) and the Triton-resistant fraction (pellet). The pellet was resuspended in 1% SDS lysis buffer (75 mM NaCl, 24 mM EDTA, pH 8.0) and incubated for 1 h at room temperature. The sample was centrifuged at 15,000 ***g*** for 10 min to separate the SDS-soluble fraction (supernatant) and SDS-resistant fraction (pellet). The pellet was dissolved in sample buffer (63 mM Tris-HCl, 2% SDS, 10% glycerol and 0.003% Bromophenol Blue) and boiled for 5 min.

### Statistical analyses

Statistical analyses were performed using a two-tailed Student's *t*-test (*P<0.05, **P<0.01) in GraphPad Prism 6. Data represent mean±s.d.

## Supplementary Material

10.1242/develop.201724_sup1 Supplementary information

## Data Availability

All relevant data can be found within the article and its supplementary information.
