# Supplementary material for: CCDC183 is essential for cytoplasmic invagination around the flagellum during spermiogenesis and male fertility
Source: Development. 2023 Oct 30;150(21):dev201724. doi: 10.1242/dev.201724 (PMC10629680; doi:10.1242/dev.201724)
Supplement: Supplementary information [file develop-150-201724-s1.pdf]

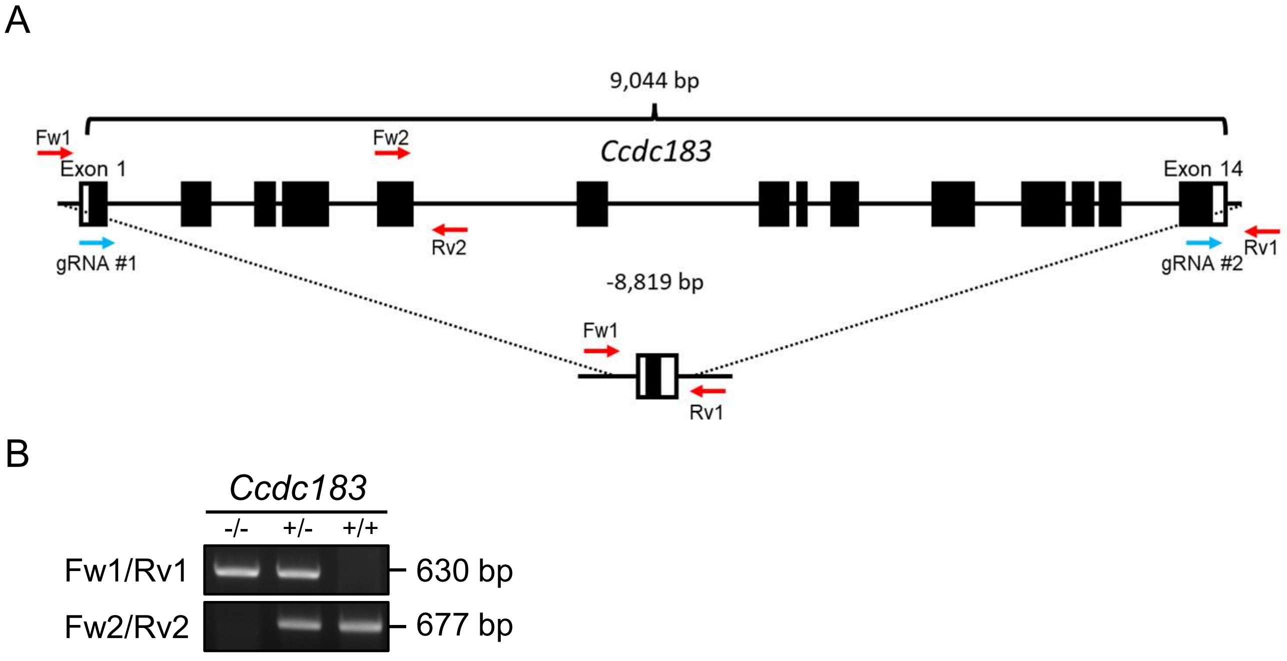

Fig. S1. Generation of *Ccdc183*-disrupted mice

(A) KO strategy for generating *Ccdc183* KO mice. Upper and bottom panels show diagrams for WT and KO alleles, respectively. Two gRNAs (blue arrows) were designed to target exon 1 and 14. Fw1 and Fw2 are forward primers for genotyping. Rv1 and Rv2 are reverse primers for genotyping. Our study generated a *Ccdc183* KO mouse line with an 8,819-bp deletion. (B) Genotyping of *Ccdc183* KO mutant mice. Fw1/Rv1 and Fw2/Rv2 primers in Fig. S1A were used.

Development • Supplementary information

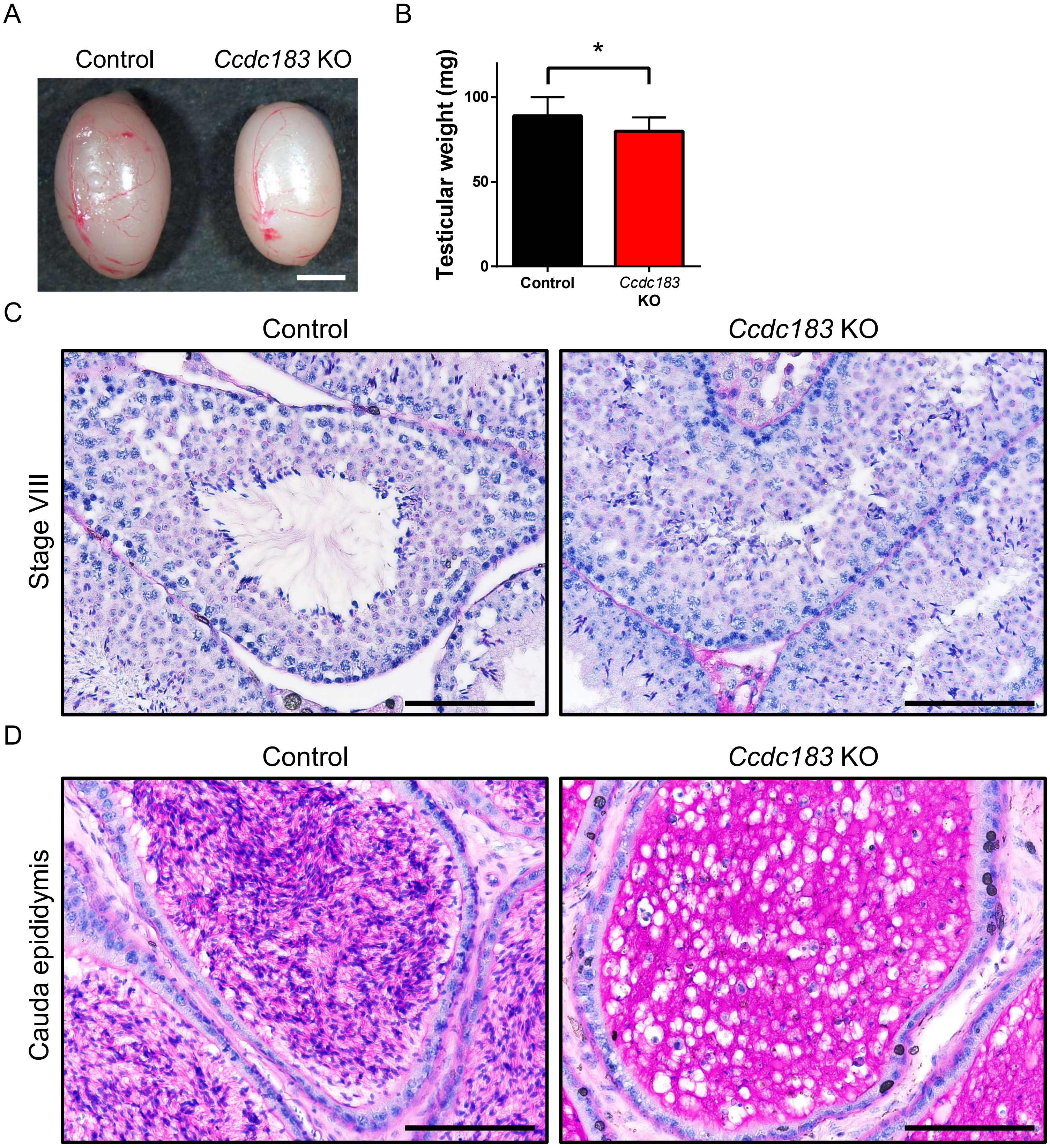

Fig. S2. *Ccdc183*-disrupted male mice have fewer spermatozoa

(A) Gross morphology of control and *Ccdc183* KO testes. Scale bars: 2.0 mm. (B) Testis weight of control and *Ccdc183* KO mice. Average weight of testis in control = 89.0 ± 11.0 mg; *Ccdc183* KO = 79.8 ± 8.3 mg. *P* = 0.0313. (C) PAS staining of testis sections of a stage VIII seminiferous tubule. Condensed sperm heads were observed near the lumen of the tubule, but sperm tails were hardly observed in *Ccdc183* KO testis. Note that the lumen of the *Ccdc183* KO seminiferous tubule has a very low volume. Scale bars: 100 μm. (D) PAS staining of cauda epididymis sections. Normal spermatozoa are difficult to observe in *Ccdc183* KO cauda epididymis. Scale bars: 100 μm.

Development • Supplementary information

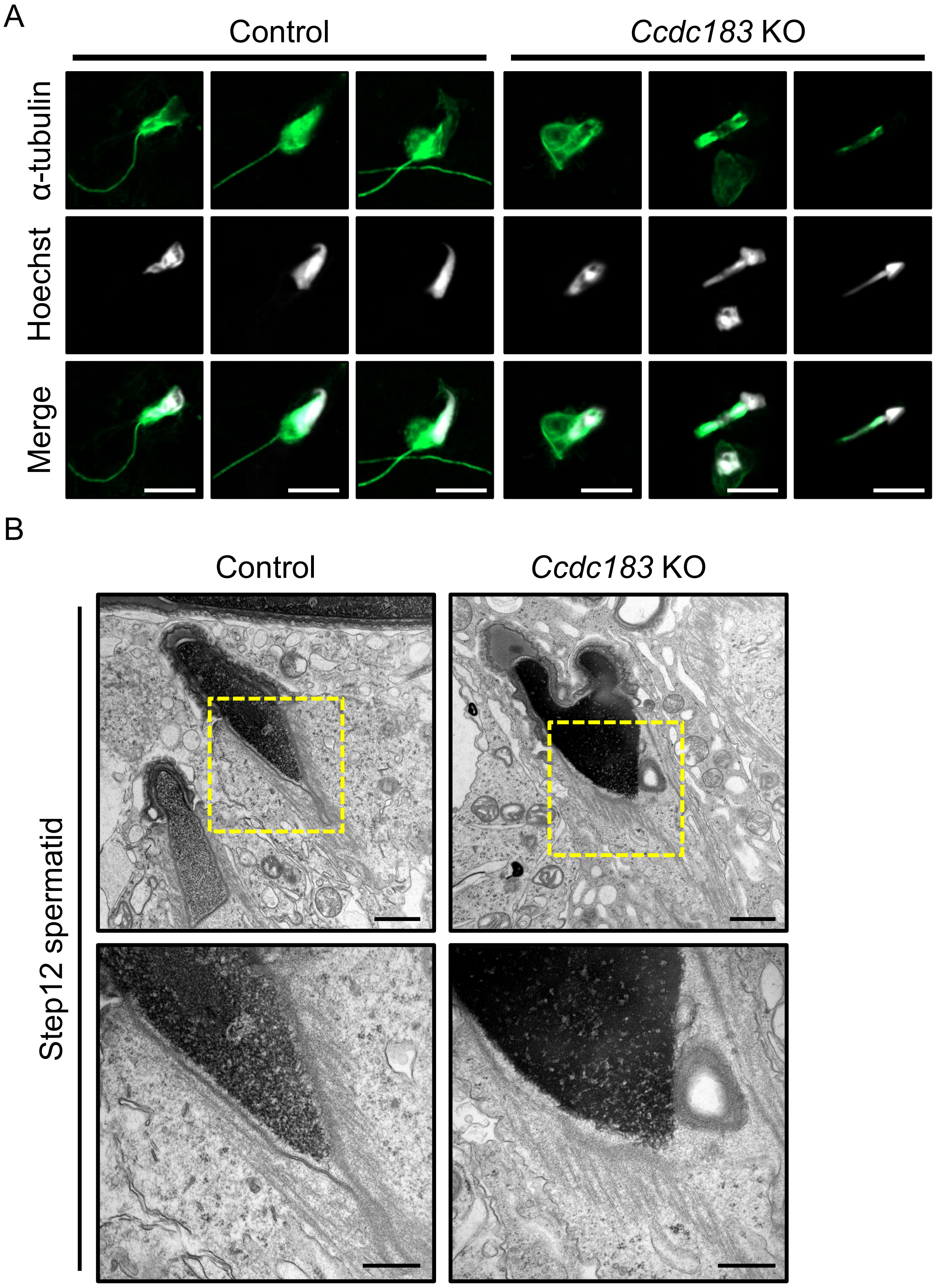

Fig. S3. *Ccdc183*-disrupted sperm heads exhibit abnormal shapes due to abnormal sculpting by the manchette

(A) Immunofluorescence analysis of spermatids from control and *Ccdc183* KO mice labeled with an antibody against α-tubulin (green). Head elongation is shown progressively from left to right based on nuclear and manchette shapes. *Ccdc183* KO spermatids exhibit abnormal manchette morphology, which leads to abnormal sperm head shape. Scale bars: 10 µm. (B) Ultrastructural images of step 12 spermatids analyzed by TEM. The bottom figures are magnified images of the boxed areas of the top figures. Although *Ccdc183* KO spermatids form abnormal manchettes, the manchette microtubules appear normal. Scale bars: 1.0 µm (top), 500 nm (bottom).

Development • Supplementary information

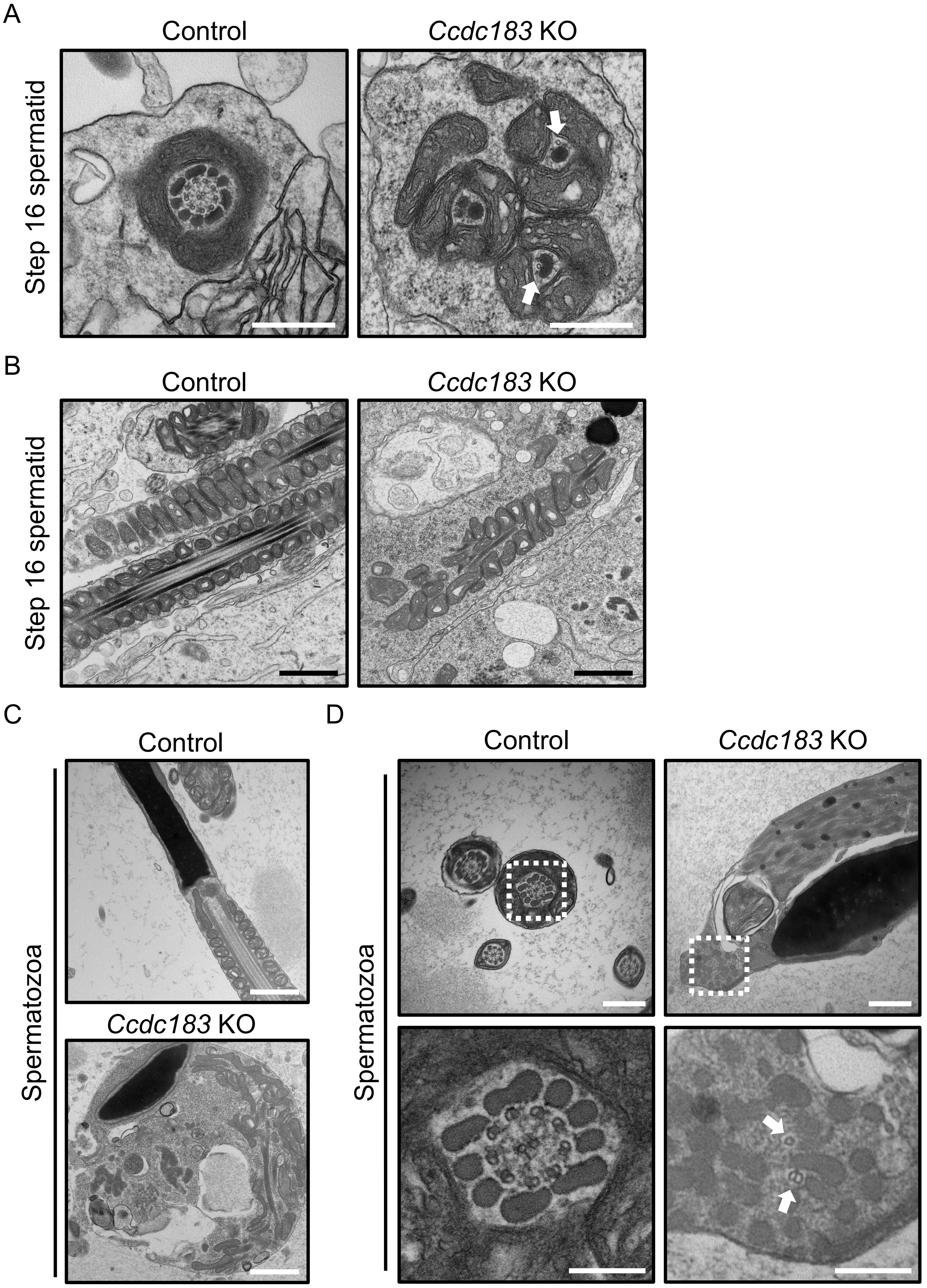

Development • Supplementary information

Fig. S4. *Ccdc183*-disrupted male mice have collapsed axonemal microtubules during spermiogenesis
(A) Ultrastructural images of step 16 spermatids analyzed by TEM. Axoneme structures were collapsed in *Ccdc183* KO spermatids and disintegrated microtubules were observed (arrows). Scale bars: 500 nm. (B) Ultrastructural images of step 16 spermatids. The mitochondrial sheath and outer dense fibers were not correctly localized around the axoneme, and axoneme structure was not observed in *Ccdc183* KO spermatids. Scale bars: 1.0 µm. (C) Ultrastructural images of spermatozoa in cauda epididymis analyzed by TEM. *Ccdc183* KO spermatozoa have a huge cytoplasm with abundant cytoplasmic contents including electron-dense materials. Scale bars: 1.0 µm. (D) Ultrastructural images of spermatozoa in cauda epididymis analyzed by TEM. The bottom figures are magnified images of the boxed areas of the top figures. *Ccdc183* KO spermatozoa lacked normal axonemes, but scattered microtubules were rarely observed (arrows). Scale bars: 500 nm (top), 200 nm (bottom).

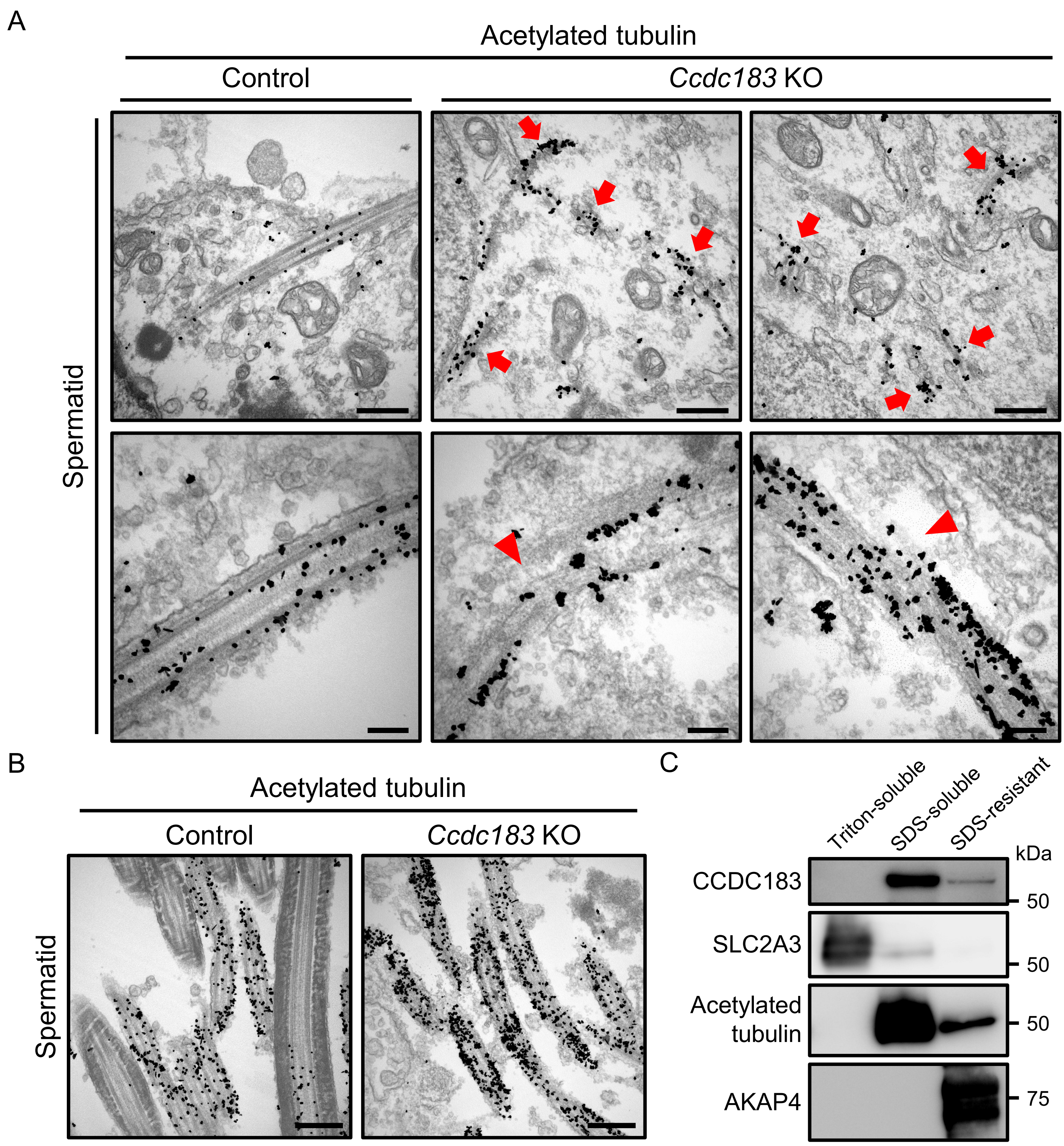

Fig. S5. Axoneme within the cytoplasm collapses during spermiogenesis in *Ccdc183* KO spermatids

(A) Detection of immunolabeled acetylated-tubulin in spermatids observed by TEM. Scattered microtubule bundles (arrows) and broken axonemal microtubules (arrowheads) were observed within the spermatid cytoplasm. Scale bars: 500 nm (top), 200 nm (bottom). (B) Detection of immunolabeled acetylated-tubulin in spermatids observed by TEM. Normal axonemal microtubules were observed within the flagellum, but no accessory structures were observed around the axoneme in *Ccdc183* KO spermatids. Note that axonemal microtubules with accessory structures in control are hardly labeled with gold particles. Scale bars: 500 nm. (C) Western blot analysis using fractionated proteins of mouse spermatozoa. CCDC183 was mainly found in the SDS-soluble fraction, which may be associated with the axonemal microtubules. SLC2A3, acetylated tubulin, and AKAP4 were detected as markers for Triton-soluble, SDS-soluble, and SDS-resistant fractions, respectively.

Development • Supplementary information

Table S1. Primer list

Purpose	Primer sequence	Annealing temperature (°C)	Cycles
mouse *Ccdc183* RT-PCR	Fw: AACTGCACAGGAAGGGTGAG<br>Rv: CAGTAGTTGGCCACCAGGTT	60	35
mouse *Actb* RT-PCR	Fw: CATCCGTAAAGACCTCTATGCCAAC<br>Rv: ATGGAGCCACCGATCCACA	60	35
Ccdc183 WT allele genotyping	Fw: AGATCACCACCAGCCAGAAC<br>Rv: CTGCCCTGAAGCACTTTCTC	65	40
Ccdc183 KO allele genotyping	Fw: GTCCAAGGATATGGGGAGGT<br>Rv: GCTAAAAGGGACACGTAGC	65	40

Table S2. Antibody list

Immunoblot analysis

Antibody	Clone No.	Host species	Catalog No.	Company	Dilution
anti-acetylated tubulin	6-11B-1	Mouse	T7451	Sigma	1:2000
anti-AKAP3	polyclonal	Rabbit	13907-1-AP	proteintech	1:1000
anti-AKAP82 (AKAP4)	Clone 25	Mouse	611564	BD biosciences	1:5000
anti-CCDC183	polyclonal	Rabbit	-	In house	1:500
anti-DNAI2	polyclonal	Rabbit	17533-1-AP	proteintech	1:1000
anti-DNALI1	polyclonal	Rabbit	17601-1-AP	proteintech	1:1000
anti-DRC3	polyclonal	Rabbit	HPA036040	Atlas Antibodies	1:500
anti-Histone H3	1G1	Mouse	sc-517576	Santacruz	1:500
anti-IZUMO1	KS064-125	Rat	-	In house	1:1000
anti-ODF2	polyclonal	Rabbit	12058-1-AP	proteintech	1:500
anti-RSPH6A	polyclonal	Rabbit	-	In house	1:1000
anti-SLC2A3	KS64-10	Rat	-	In house	1:1000

Immunofluorescence analysis

Antibody	Clone No.	Host species	Catalog No.	Company	Dilution for testis	Dilution for sperm
anti-acetylated tubulin	6-11B-1	Mouse	T7451	Sigma	1:500	1:500
anti-AKAP3	polyclonal	Rabbit	13907-1-AP	proteintech	1:1000	-
anti-α-tubulin	B-5-1-2	Mouse	T6074	abcam	1:2000	-
anti-ODF2	polyclonal	Rabbit	12058-1-AP	proteintech	1:200	-
anti-TOMM20	EPR15581-5	Rabbit	ab186735	abcam	-	1:100

Immunoelectron microscopy analysis

Antibody	Clone No.	Host species	Catalog No.	Company	Dilution
anti-acetylated tubulin	6-11B-1	Mouse	T7451	Sigma	1:150
anti-AKAP3	polyclonal	Rabbit	13907-1-AP	proteintech	1:150
anti-ODF2	polyclonal	Rabbit	12058-1-AP	proteintech	1:150
