# Supplementary material for: CCDC183 is essential for cytoplasmic invagination around the flagellum during spermiogenesis and male fertility
Source: Development. 2023 Oct 30;150(21):dev201724. doi: 10.1242/dev.201724 (PMC10629680; doi:10.1242/dev.201724)
Supplement: Supplementary file 1 [file develop-150-201724_review_history.pdf]

CCDC183 is essential for cytoplasmic invagination around the flagellum during spermiogenesis and male fertility

Keisuke Shimada and Masahito Ikawa
DOI: 10.1242/dev.201724

Editor: Haruhiko Koseki

Review timeline

Original submission:	21 February 2023
Editorial decision:	30 March 2023
First revision received:	1 August 2023
Editorial decision:	18 September 2023
Second revision received:	26 September 2023
Accepted:	3 October 2023

Original submission

First decision letter

MS ID#: DEVELOP/2023/201724

MS TITLE: CCDC183 is essential for proper formation of both the fibrous sheath and outer dense fibers in spermiogenesis

AUTHORS: Keisuke Shimada and Masahito Ikawa

I have now received all the referees' reports on the above manuscript, and have reached a decision. The referees' comments are appended below, or you can access them online: please go to BenchPress and click on the 'Manuscripts with Decisions' queue in the Author Area.

As you will see, the referees express considerable interest in your work, but have some significant criticisms and recommend a substantial revision of your manuscript before we can consider publication. If you are able to revise the manuscript along the lines suggested, which may involve further experiments, I will be happy receive a revised version of the manuscript. Your revised paper will be re-reviewed by one or more of the original referees, and acceptance of your manuscript will depend on your addressing satisfactorily the reviewers' major concerns. Please also note that Development will normally permit only one round of major revision. If it would be helpful, you are welcome to contact us to discuss your revision in greater detail. Please send us a point-by-point response indicating your plans for addressing the referee's comments, and we will look over this and provide further guidance.

Please attend to all of the reviewers' comments and ensure that you clearly highlight all changes made in the revised manuscript. Please avoid using 'Tracked changes' in Word files as these are lost in PDF conversion. I should be grateful if you would also provide a point-by-point response detailing how you have dealt with the points raised by the reviewers in the 'Response to Reviewers' box. If you do not agree with any of their criticisms or suggestions please explain clearly why this is so.

Reviewer 1

Advance summary and potential significance to field

© 2023. Published by The Company of Biologists under the terms of the Creative Commons Attribution License (https://creativecommons.org/licenses/by/4.0/).

The authors generated Ccdc183 KO mice and discovered that the absence of the protein induces male infertility with morphological and motility defects in spermatozoa. The fibrous sheath and outer dense fibers could not localize to their proper positions during spermiation. They conclude that that CCDC183 plays a role in the proper localization of both the fibrous sheath and outer dense fibers around the axoneme. This work unveiled that CCDC183 links the axoneme with accessory structures such as the fibrous sheath and outer dense fibers for proper spermiogenesis.

Comments for the author

he study is straight forward. The quality of the data is excellent.
However, the overall presentation is descriptive, there is no mechanismic studies to reveal how the protein plays a role in the formation of the fibrous sheath and ODF.
The authirs showed the AKAP4 western blot result. Actually the AKAP4 is processed fromn the full length pro-AKAP4. The authiors should also examine the expression of the pro-AKAP4 in the KO mice.

<u>Reviewer 2</u>

Advance summary and potential significance to field

This is an interesting paper that has the potential to make a valuable contribution to the literature including providing mechanism of action to explain the prior association between CCDC183 mutations and human male infertility. As written however, the paper is poorly resolved. I encourage the authors to step back and think about why the structural defects are occurring. The EM data is very nice. I have no concerns about the quality of the data – only the interpretation.

Specifically, the defects could be due to a failure of accessory structure attachment to the microtubules of the axoneme OR it would be due to defects in proteins delivery within the ciliary compartment OR it could be due to a failure of accessory protein entry into the cilia compartment in the first place. What experiment could be done to tease these 3 processes apart.

Comments in the order outlined in the paper

Line 116-8: slightly ambiguous wording. The mitochondrial sheath does not attach to the axoneme, so the link is not clear. Given what is described in the following sentences, I would be tempted to just delete this sentence.
Actually, this whole section would do with some tightening up. I'm not questioning the results but rather the wording. It might be helpful for non-expert readers if the text was written to reflect the biological chronological order (early step spermatids to late step spermatids). The switching between steps and stages is confusing, in part because stage is being used loosely. I recommend sticking to steps. A sentence at the start of the section explaining what a stage and a step is might also be useful. Ditto, it would help if the language was more precise e.g. axoneme microtubules rather than just microtubules (as there are numerous types).

Line 125: abnormal tails rather than disintegrated microtubules. Disintegrated suggests complete destruction. Lost patency may be a more accurate term. Or were damaged, or similar.

The sloughed germ cells seen in Fig S2 are not mentioned. This would contribute to the reduced testis weight.

Paragraph starting line 135 – I don't understand this experiment. If there are fewer elongating spermatids (and round germ cells are being lost by sloughing) how is this comparison meaningful? How was cell content normalised? This is a major criticism.

Fig 4C contains interesting data. The authors have suggested that this is a failure of the FS to elongate from the tip of the axoneme back towards the tail.
Alternatively, this could be a failure of FS (AKAP3) to enter the ciliary compartment. Has this been considered? As protein is present at the head tail coupling apparatus, this might be more likely.

© 2023. Published by The Company of Biologists under the terms of the Creative Commons Attribution License (https://creativecommons.org/licenses/by/4.0/).

This doesn't change the conclusion that no elongation occurred. Fig. 5 could also support this hypothesis – I note it's not a complete block, but notable. Very nice EM images.

Alternatively these defects could be due to defects in the modified form of IFT described in Pleuger et al PMID: 32318721.

Lines 175: please check the timing of the appearance of fibrous sheath components during spermiogenesis. Step 2 is too early based on my recollection. What species are you referring to?

Ditto you have not shown that CCDC183 is an axoneme associated protein.

Comments for the author

As outlined above

Reviewer 3

Advance summary and potential significance to field

In this manuscript, Shimada and Ikawa generate and characterize CCDC183-knockout mice. They find that CCDC183-knockout mice are infertile due to abnormal sperm structure and motility. They show that structural defects of the flagellum are particularly severe and include malformation of the axoneme and associated accessory structures (the outer dense fibers and the fibrous sheath).

Morphogenesis of the sperm flagellum is immensely complex, yet many aspects of the process remain poorly understood at the molecular level. This work contributes to our knowledge of this phenomenon by identifying a protein involved in proper formation of the sperm flagellum. This work will be of interest to researchers working on sperm, spermiogenesis, and cilia.

Comments for the author

Some conclusions as written are not sufficiently supported by the data, and revisions are therefore necessary.

In particular, the authors state that CCDC183 "links" accessory structures (ODFs and fibrous sheath) to the axoneme. In their model, the axoneme forms correctly in early stages of spermiogenesis, but the lack of CCDC183 means that the accessory structures do not correctly localize around the axoneme, causing microtubules to become fragile and to ultimately disintegrate in later stages of spermiogenesis.

This conclusion needs to either be strengthened with additional data or re-phrased to account for the uncertainty still present. In general, better quantification of the TEM data would help clarify the results. How common were normal versus abnormal axonemes in KO spermatids (at both early versus late stages)? For example, the claim that the 9+2 axoneme forms properly in early spermiogenesis is supported by a single TEM image shown in Fig. 3A, but how many TEM images is this statement based on?

Likewise, the spermatozoon shown in Fig. 4B has microtubules with spacing consistent with what is expected of a typical 9+2 axoneme, but the KO sperm shown in Figure 3B and the late-stage spermatids shown in Fig. S3C have completely disorganized microtubules, with no semblance of the 9+2 architecture. Again, how often do the authors see sperm with fully-formed axonemes (but no accessory structures) versus sperm with disorganized microtubules?

With the current data, it seems that CCDC183 may be involved in forming the axoneme as a whole (and not just in the localization of accessory structures after the axoneme has formed). For instance, in the CCDC183-KO spermatid shown in Fig. 5A, there seems to only be a central pair present, with no sign of any doublet microtubules extending from the distal centriole. If the 9+2 axoneme had indeed formed correctly and subsequently fractured, one would expect to see broken doublet microtubules present around the central pair, which does not seem to be the case. Indeed,

© 2023. Published by The Company of Biologists under the terms of the Creative Commons Attribution License (https://creativecommons.org/licenses/by/4.0/).

do the authors see any evidence that axonemal microtubules become fragmented after they are formed (i.e. longitudinal sections with broken doublet microtubules)?

In addition, the authors observed head shape abnormalities and suggest malfunction of the manchette (lines 108-109). These observations are only based on histology staining with PAS. The authors should provide TEM images focusing on spermatids (early and late) heads and manchette. Furthermore, manchette microtubules are very different from axoneme microtubules but throughout the manuscript the authors refer to them as similar. This needs to be fixed as it has important implications on the conclusions. Are the authors suggest CCDC183 'an axonemal associated protein' (line 172) or microtubule associated protein in more general? On this note, even 'axonemal microtubules' is a general term because the doublets and singlets in the axoneme are different and the singlets in the endpiece are not the same as the singlets in the axoneme. So the authors should be more specific in their conclusions (lines 190-193).

It will help a lot if the authors can add immunogold labeling of CCDC183 on WT spermatids and spermatozoa, to support their claims that CCDC183 binds axoneme and manchette.

Below are some specific comments:

Line 50-51: "The accessory structures of the axoneme contain inner dynein arms…"
- It is probably best to re-word this as the term "accessory structures" is typically used to refer to the ODFs and the fibrous sheath. A suggestion would be:
"Protein complexes bound to axonemal microtubules include…"

Lines 29 and 54: 'the endpiece contains no accessory structures'
- The endpiece in mouse also does not contain axoneme, needs to be more accurate.

Line 89-90: "Even spermatozoa that appear normal have abnormalities when looked at closely such as flagellar thickness"
- It would help if the authors could show data for this, for example zoomed-in views comparing WT and KO cells, and ideally some quantification.

Line 105-107: "As proper manchette formation and removal are crucial for proper sperm head development, we stained manchette using anti-α-tubulin antibody and found that there were abnormalities in manchette formation in KO spermatids (Fig. S3A)."
- There are clearly defects in the manchette, but there also appear to be structural abnormalities in the flagellum itself.
- Have the authors examined the ultrastructure of the manchette in KO sperm?

Line 119-120: "We could observe normal 9+2 microtubule structures in step 6 KO spermatids (Fig. 3A), which is an early stage of spermiogenesis."
- Previous TEM data suggests that ODF form in close association with their respective DMTs, starting out as thin fibers that then expand later in spermiogenesis. In the TEM image of a CCDC183 KO axoneme shown in Figure 3A, it looks like the doublet microtubules show signs of early ODF formation specifically electron-dense protrusions close to the outer junction. How does this fit in to the authors' model?

Line 122-123: "In addition, fragmented microtubules were found in the KO spermatozoa collected from the cauda epididymis (Fig. 3B)."
- In Figure 3B, the doublet microtubule indicated with an arrow looks like it is attached to an ODF?

Line 132-133: "CCDC183 protein was mainly extracted from SDS-soluble fraction (Fig. 3D), which indicates that CCDC183 is associated with the axoneme."
- It is worth mentioning that at least some CCDC183 remains SDS-insoluble which suggests that some of the protein may be associated with the accessory structures themselves.

Line 178-180: "We observed CCDC183 KO testis carefully to find proper 9+2 microtubule structures, and could only find axonemes without any accessory structures such as outer dense fibers and/or fibrous sheath (see Fig. 3A)."

© 2023. Published by The Company of Biologists under the terms of the Creative Commons Attribution License (https://creativecommons.org/licenses/by/4.0/).

- Many of the TEM images shown do not have properly-formed axonemes (only disorganized microtubules), so perhaps it is better to rephrase this statement.

Methodology
- It would be very helpful to the readers if the authors could at least briefly describe all experimental procedures in this manuscript (and not just refer to previous papers). For instance, how head and tails were separated?

Lastly, the results jump back and for from spermatids (different stages) and spermatozoa. To add to the clarity the authors should separate (not in the same figure, e.g., Fig. 3) between results on spermatids and results on spermatozoa and spermatids results should be sorted from early to late spermatids. Finally, the authors should include a schematic summarizing their observations from the KO and the function of CCDC183 proposed.

First revision

Author response to reviewers' comments

Reviewer 1 Advance Summary and Potential Significance to Field:
The authors generated Ccdc183 KO mice and discovered that the absence of the protein induces male infertility with morphological and motility defects in spermatozoa. The fibrous sheath and outer dense fibers could not localize to their proper positions during spermiation. They conclude that that CCDC183 plays a role in the proper localization of both the fibrous sheath and outer dense fibers around the axoneme. This work unveiled that CCDC183 links the axoneme with accessory structures such as the fibrous sheath and outer dense fibers for proper spermiogenesis.

Reviewer 1 Comments for the Author: The study is straight forward. The quality of the data is excellent. However, the overall presentation is descriptive, there is no mechanistic studies to reveal how the protein plays a role in the formation of the fibrous sheath and ODF. The authors showed the AKAP4 western blot result. Actually, the AKAP4 is processed from the full length pro-AKAP4. The authors should also examine the expression of the pro-AKAP4 in the KO mice.

Thank you for your comments. After confirming the reviewers' comments, we restudied the mechanism of abnormal sperm morphology again, and found that CCDC183 is essential for cytoplasmic invagination during spermiogenesis, and the disruption of accessory structures was an indirect phenotype. Due to these findings, we needed to revise a significant portion of our paper. But we believe this modification increases the quality of this paper. Sorry to bother you, but I would like to ask you to evaluate this article again.
We checked the expression of the pro-AKAP4 and found the expression level was not changed (Fig. 4B). This result may indicate that there is a problem in the cleavage of precursor protein in the KO mice.

***** Reviewer 2 Advance Summary and Potential Significance to Field:
This is an interesting paper that has the potential to make a valuable contribution to the literature including providing mechanism of action to explain the prior association between CCDC183 mutations and human male infertility. As written however, the paper is poorly resolved. I encourage the authors to step back and think about why the structural defects are occurring. The EM data is very nice. I have no concerns about the quality of the data - only the interpretation.
Thank you for your comments. The revised manuscript focuses on understanding the reasons behind structural defects in KO, as per the reviewer's comment.

Specifically, the defects could be due to a failure of accessory structure attachment to the microtubules of the axoneme OR it would be due to defects in proteins delivery within the ciliary compartment OR it could be due to a failure of accessory protein entry into the cilia compartment in the first place. What experiment could be done to tease these 3 processes apart.
I think this comment contain very deep implications. We did not consider flagellum formation in

© 2023. Published by The Company of Biologists under the terms of the Creative Commons Attribution License (https://creativecommons.org/licenses/by/4.0/).

depth in our first draft. However, after reading this comment, we learned the need to know it in depth. Additional experiments (Fig. 4D) revealed that the FS protein could not enter the flagellar compartment or cannot be transported through the IFT in CCDC183-deficient sperm. But we could not determine which hypothesis is correct (Lines 232-237).

Comments in the order outlined in the paper

Line 116-8: slightly ambiguous wording. The mitochondrial sheath does not attach to the axoneme, so the link is not clear. Given what is described in the following sentences, I would be tempted to just delete this sentence. Actually, this whole section would do with some tightening up. I'm not questioning the results but rather the wording. It might be helpful for non-expert readers if the text was written to reflect the biological chronological order (early step spermatids to late step spermatids).
Thank you for your comments. Due to the big changes of the contents, this paragraph (Lines 128-140) has been changed. The revised manuscript showed that CCDC183 deficiency causes axonemal collapse due to abnormal cytoplasmic invagination around the flagellum. We apologize for the inconvenience, but we would appreciate a re-evaluation of the revised argument.

The switching between steps and stages is confusing, in part because stage is being used loosely. I recommend sticking to steps. A sentence at the start of the section explaining what a stage and a step is might also be useful. Ditto, it would help if the language was more precise e.g. axoneme microtubules rather than just microtubules (as there are numerous types).
According to the reviewer's comments, we recognized the need for the time sequence of spermatogenesis, and unified using "step" to represent spermatid status.

Line 125: Abnormal tails rather than disintegrated microtubules. Disintegrated suggests complete destruction. Lost patency may be a more accurate term. Or were damaged, or similar.
Thank you for your suggestion. As it is true that we could not reveal the sperm tail destruction from these figures, we changed the term. We just wrote "microtubules were scattered" (Lines 136- 138).

The sloughed germ cells seen in Fig S2 are not mentioned. This would contribute to the reduced testis weight.
Thank you for your suggestion. We added this explanation in lines 111-112.

Paragraph starting line 135 – I don't understand this experiment. If there are fewer elongating spermatids (and round germ cells are being lost by sloughing) how is this comparison meaningful? How was cell content normalized? This is a major criticism.
We performed western blot analysis using testicular proteins (Fig. 4B). Total protein amount between control and KO was normalized. We added the result of α-tubulin. These WB results indicate that the amount of protein forming axonemal microtubules itself has not changed, but the amount of stable microtubules has decreased, which reflects abnormal axonemal formation (Lines 171-173).

Fig 4C contains interesting data. The authors have suggested that this is a failure of the FS to elongate from the tip of the axoneme back towards the tail. Alternatively, this could be a failure of FS (AKAP3) to enter the ciliary compartment. Has this been considered? As protein is present at the head tail coupling apparatus, this might be more likely. This doesn't change the conclusion that no elongation occurred. Fig. 5 could also support this hypothesis – I note it's not a complete block, but notable. Very nice EM images.
To clarify the questions, we performed immuno-EM analysis using anti-AKAP3 antibody (Fig. 4D). We could not find AKAP3 with in the flagellum, which suggests that FS proteins could not enter the flagellum compartment (Lines 185-187). In addition, we could not deny the possibility of protein delivery failure via IFT, so we added this possibility to the discussion section (Lines 232-237).

Alternatively, these defects could be due to defects in the modified form of IFT described in Pleuger et al PMID: 32318721.
Thank you for the helpful reference. After reading this paper, we came up with new experiments. But it was unclear whether protein entry into the flagellum compartment or protein delivery via IFT failed in KO mice (Lines 233-235).

© 2023. Published by The Company of Biologists under the terms of the Creative Commons Attribution License (https://creativecommons.org/licenses/by/4.0/).

Lines 175: please check the timing of the appearance of fibrous sheath components during spermiogenesis. Step 2 is too early based on my recollection. What species are you referring to?
Irons and Clermont performed radioautographic labeling for the fibrous sheath. They found the longitudinal columns form the distal end of the principal piece in rat step 2 spermatids. In our first draft, it was written as if the FS was completed in step 2. Therefore, we changed it to say that the precursor of FS starts to express in step 2 (Lines 61 and 223).
Irons MJ & Clermont Y (1982) Kinetics of fibrous sheath formation in the rat spermatid. Am. J. Anat. 165(2):121-130.

Ditto you have not shown that CCDC183 is an axoneme associated protein.
CCDC183 is found in the SDS-soluble fraction (Fig. S5C), which is the same fraction as axonemal microtubules. But we know it is not sufficient to say CCDC183 is an axonemal protein. Therefore, we have moderated our expression regarding the question of whether or not CCDC183 belongs to the axonemal system (Lines 159-161).

***** Reviewer 3 Advance Summary and Potential Significance to Field:
In this manuscript, Shimada and Ikawa generate and characterize CCDC183-knockout mice. They find that CCDC183-knockout mice are infertile due to abnormal sperm structure and motility. They show that structural defects of the flagellum are particularly severe and include malformation of the axoneme and associated accessory structures (the outer dense fibers and the fibrous sheath).
Morphogenesis of the sperm flagellum is immensely complex, yet many aspects of the process remain poorly understood at the molecular level. This work contributes to our knowledge of this phenomenon by identifying a protein involved in proper formation of the sperm flagellum. This work will be of interest to researchers working on sperm, spermiogenesis, and cilia.

Reviewer 3 Comments for the Author: Some conclusions as written are not sufficiently supported by the data, and revisions are therefore necessary.

In particular, the authors state that CCDC183 “links” accessory structures (ODFs and fibrous sheath) to the axoneme. In their model, the axoneme forms correctly in early stages of spermiogenesis, but the lack of CCDC183 means that the accessory structures do not correctly localize around the axoneme, causing microtubules to become fragile and to ultimately disintegrate in later stages of spermiogenesis.
This conclusion needs to either be strengthened with additional data or re-phrased to account for the uncertainty still present. In general, better quantification of the TEM data would help clarify the results. How common were normal versus abnormal axonemes in KO spermatids (at both early versus late stages)? For example, the claim that the 9+2 axoneme forms properly in early spermiogenesis is supported by a single TEM image shown in Fig. 3A, but how many TEM images is this statement based on?
Likewise, the spermatozoon shown in Fig. 4B has microtubules with spacing consistent with what is expected of a typical 9+2 axoneme, but the KO sperm shown in Figure 3B and the late-stage spermatids shown in Fig. S3C have completely disorganized microtubules, with no semblance of the 9+2 architecture. Again, how often do the authors see sperm with fully-formed axonemes (but no accessory structures) versus sperm with disorganized microtubules?
Thank you for your comments. First of all, we must admit that we have drawn erroneous conclusions from the data obtained. Our new data revealed that if axonemal microtubules remain intact, accessory structure proteins could localize properly (Fig. 4D and 5C). This means CCDC183 does NOT link accessory structures to the axoneme.
As the reviewer pointed out, we attempted to quantify the presence of abnormal axonemal microtubules by TEM. However, it was difficult to tell whether collapsed microtubules were microtubules or not, so quantification was impossible. Because it is impossible to count collapsed microtubules, we immunolabeled microtubule-associated protein and observed by TEM. When we compare the data in Fig. S5A and S5B, we could see the difference in microtubule stability within the cytoplasm and within the flagellum. Although we believe that axonemes in the cytoplasm are 100% abnormal and that axonemes in the flagella are 100% abnormal, we cannot say with 100% certainty that there are no abnormalities based on the structure of the longitudinal sections (Fig. S5B), and since we can rarely detect cleanly observable transverse sections like Fig. 3A, we determined that quantitative observation by TEM was not possible.

© 2023. Published by The Company of Biologists under the terms of the Creative Commons Attribution License (https://creativecommons.org/licenses/by/4.0/).

With the current data, it seems that CCDC183 may be involved in forming the axoneme as a whole (and not just in the localization of accessory structures after the axoneme has formed). For instance, in the CCDC183-KO spermatid shown in Fig. 5A, there seems to only be a central pair present, with no sign of any doublet microtubules extending from the distal centriole. If the 9+2 axoneme had indeed formed correctly and subsequently fractured, one would expect to see broken doublet microtubules present around the central pair, which does not seem to be the case. Indeed, do the authors see any evidence that axonemal microtubules become fragmented after they are formed (i.e. longitudinal sections with broken doublet microtubules)?
We found this comment correct. After the Immuno-EM analysis, we found that many microtubule bundles with breaks were within the KO cytoplasm (Fig. S5A). But axonemes with only central microtubules in clear cross-section were not observed. Thanks to the immuno-EM observation, however, we realized that axonemes within the flagellum have microtubule stability, but not axonemes within the cytoplasm. We are grateful for this comment because this discovery continues to help us notice abnormalities in cytoplasmic invagination.

In addition, the authors observed head shape abnormalities and suggest malfunction of the manchette (lines 108-109). These observations are only based on histology, staining with PAS. The authors should provide TEM images focusing on spermatids (early and late) heads and manchette.
We checked manchette microtubules in KO spermatid and found no problems in the structure (Fig. S3B).

Furthermore, manchette microtubules are very different from axoneme microtubules but throughout the manuscript the authors refer to them as similar. This needs to be fixed as it has important implications on the conclusions. Are the authors suggest CCDC183 'an axonemal associated protein' (line 172) or microtubule associated protein in more general? On this note, even 'axonemal microtubules' is a general term because the doublets and singlets in the axoneme are different and the singlets in the endpiece are not the same as the singlets in the axoneme. So, the authors should be more specific in their conclusions (lines 190-193).
Thank you for your perceptive remarks. As response to reviewer 2 comment, we should not conclude CCDC183 is an axonemal associated protein (Lines 159-161). In the draft manuscript, we had a poor understanding of microtubules such as equating axonemal microtubules and manchette microtubules. In response to these reviewers' comments, we performed new experiments such as immuno-EM analysis and reconsidered the conclusions. As a result, the paper content was very different from the first draft. But we believe the quality has definitely improved. We apologize for the inconvenience, but we ask that you evaluate the revised paper again.

It will help a lot if the authors can add immunogold labeling of CCDC183 on WT spermatids and spermatozoa, to support their claims that CCDC183 binds axoneme and manchette.
Unfortunately, specific immunolabeling of CCDC183 was impossible. Therefore, the localization of CCDC183 remains unknown.

Below are some specific comments:
Line 50-51: "The accessory structures of the axoneme contain inner dynein arms…"
-It is probably best to re-word this as the term "accessory structures" is typically used to refer to the ODFs and the fibrous sheath. A suggestion would be: "Protein complexes bound to axonemal microtubules include…"
Thank you for your comments. We modified it to "macromolecular complexes" (Line 48).

Lines 29 and 54: 'the endpiece contains no accessory structures'
-The endpiece in mouse also does not contain axoneme, needs to be more accurate.
I checked several papers about endpiece and confirmed that endpiece contains central pair and outer doublet. Therefore, I think this sentence should be correct.
Russell L, Ettlin R, Sinha Hikim A, & Clegg E (1990) Histological and histopathological evaluation of the testis. 1990. (Cache River Press).
Turner RM. Moving to the beat: a review of mammalian sperm motility regulation. Reprod Fertil Dev. 2006;18(1-2):25-38. doi: 10.1071/rd05120. PMID: 16478600.

Line 89-90: "Even spermatozoa that appear normal have abnormalities when looked at closely such as flagellar thickness"

© 2023. Published by The Company of Biologists under the terms of the Creative Commons Attribution License (https://creativecommons.org/licenses/by/4.0/).

-It would help if the authors could show data for this, for example zoomed-in views comparing WT and KO cells, and ideally some quantification.
Thank you for your comments. We added enlarged figures (Fig. 1D) and quantified data (Fig. 1E).

Line 105-107: “As proper manchette formation and removal are crucial for proper sperm head development, we stained manchette using anti-α-tubulin antibody and found that there were abnormalities in manchette formation in KO spermatids (Fig. S3A).”
-There are clearly defects in the manchette, but there also appear to be structural abnormalities in the flagellum itself.
-Have the authors examined the ultrastructure of the manchette in KO sperm?
Thank you for your good suggestion. As reviewer’s comment, there are structural abnormalities in the flagellum itself (Fig. S3A). We added new sentences (Lines 122-124, 206-207). We believe this result is caused by the axonemal microtubule collapse within the cytoplasm (Fig. S5A).
We checked ultrastructure of the manchette and found no abnormalities (Fig. S3B).

Line 119-120: “We could observe normal 9+2 microtubule structures in step 6 KO spermatids (Fig. 3A), which is an early stage of spermiogenesis.”
-Previous TEM data suggests that ODF form in close association with their respective DMTs, starting out as thin fibers that then expand later in spermiogenesis. In the TEM image of a CCDC183 KO axoneme shown in Figure 3A, it looks like the doublet microtubules show signs of early ODF formation, specifically electron-dense protrusions close to the outer junction. How does this fit in to the authors’ model?
As a result of immuno-EM analysis, both fibrous sheath and outer dense fiber proteins could not be observed in flagellar compartment. Therefore, we think these black ones observed in Fig. 3A are not precursors of accessory structures.

Line 122-123: “In addition, fragmented microtubules were found in the KO spermatozoa collected from the cauda epididymis (Fig. 3B).”
-In Figure 3B, the doublet microtubule indicated with an arrow looks like it is attached to an ODF?
Thank you for your comments. We added this information in the sentence (Line 136).

Line 132-133: “CCDC183 protein was mainly extracted from SDS-soluble fraction (Fig. 3D), which indicates that CCDC183 is associated with the axoneme.”
-It is worth mentioning that at least some CCDC183 remains SDS-insoluble, which suggests that some of the protein may be associated with the accessory structures themselves.
Thank you for your suggestion. We added this information in the sentence (Lines 161-162).

Line 178-180: “We observed CCDC183 KO testis carefully to find proper 9+2 microtubule structures, and could only find axonemes without any accessory structures such as outer dense fibers and/or fibrous sheath (see Fig. 3A).”
-Many of the TEM images shown do not have properly-formed axonemes (only disorganized microtubules), so perhaps it is better to rephrase this statement.
By taking new data, we realized that axoneme within the flagellum are intact, but not within the cytoplasm. Therefore, this sentence is inaccurate. We modified almost all discussion section.

Methodology
-It would be very helpful to the readers if the authors could at least briefly describe all experimental procedures in this manuscript (and not just refer to previous papers). For instance, how head and tails were separated?
We described procedures in detail for manchettes staining, ultrastructural analysis, and fractionation of sperm protein.

Lastly, the results jump back and for from spermatids (different stages) and spermatozoa. To add to the clarity the authors should separate (not in the same figure, e.g., Fig. 3) between results on spermatids and results on spermatozoa and spermatids results should be sorted from early to late spermatids. Finally, the authors should include a schematic summarizing their observations from the KO and the function of CCDC183 proposed.
The discussion section outlined what happens in KO spermatogenesis, separating it into the flagellum, fibrous sheath, outer dense fibers, and head morphology. In addition, we added new diagrams for flagellar formation in control and KO spermatids (Figs. 6A and 6B). We believe these

© 2023. Published by The Company of Biologists under the terms of the Creative Commons Attribution License (https://creativecommons.org/licenses/by/4.0/).

modifications will make the content more understandable for readers.

Second decision letter

MS ID#: DEVELOP/2023/201724

MS TITLE: CCDC183 is essential for cytoplasmic invagination around the flagellum during spermiogenesis and male fertility

AUTHORS: Keisuke Shimada and Masahito Ikawa

I have now received all the referees reports on the above manuscript, and have reached a decision. The referees' comments are appended below, or you can access them online: please go to BenchPress and click on the 'Manuscripts with Decisions' queue in the Author Area.

The overall evaluation is positive and we would like to publish a revised manuscript in Development, provided that the referees' comments can be satisfactorily addressed. Please attend to all of the reviewers' comments in your revised manuscript and detail them in your point-by-point response. If you do not agree with any of their criticisms or suggestions explain clearly why this is so. If it would be helpful, you are welcome to contact us to discuss your revision in greater detail. Please send us a point-by-point response indicating your plans for addressing the referees' comments, and we will look over this and provide further guidance.

Reviewer 1

Advance summary and potential significance to field

The authors addressed my major questions. Particularly, they provided the western blot result to show the AKAP4 expression, which is a strong evidence to support the failure of FS formation.

Comments for the author

I appreciate that the authors discuss the potential role of IFT in the new mouse model. However, the citation is not appropriate The current citation is a review paper. There are a number of research papers about the role of IFT in mouse sperm formation, and some have been shown to be related to FS. Those original papers should be cited.

Reviewer 2

Advance summary and potential significance to field

The authors are to be congratulated on the approach they have taken to revising this manuscript. This paper was good to start with, but it is even better after the revisions. I am confident it will make a valuable contribution to the literature. The phenotype described, and thus the function of CCDC183, is very interesting and adds an important piece to the puzzle of how sperm tails are formed.

The fact that plasma membrane docking to the basal body appears to have failed yet axonemes grow (even if abnormal) is remarkable and suggestive of the cytoplasmic axoneme growth observed in drosophila – but not usually in mammals – suggesting that CCDC183 is evolutionary important. See the paper below for a summary.

https://www.ncbi.nlm.nih.gov/pmc/articles/PMC4254545/
https://pubmed.ncbi.nlm.nih.gov/26654377/

© 2023. Published by The Company of Biologists under the terms of the Creative Commons Attribution License (https://creativecommons.org/licenses/by/4.0/).

In other germ cells, as the authors have clearly shown, the axoneme is present but the ODF and FS are absent from the ciliary lobe. I agree, that based on the current information it is not possible to determine if CCDC183 plays a role in protein entry into the ciliary compartment e.g. in the transition zone, or it plays a role in protein delivery along the axoneme/ciliary lobe e.g. via the IFT pathway. Regardless, the insights are valuable.

The discussion on the ortholog (or not) between mouse and fly CCDC183 is important.

The summary figure is helpful.

Comments for the author

I have no edits to suggest.

Reviewer 3

Advance summary and potential significance to field

In their revised manuscript, Shimada & Ikawa present new data on CCDC183-knockout mice, re-interpreting the cellular basis of the observed male infertility. Their major new conclusion is that CCDC183 knockouts are infertile due to defects occurring around the cytoplasmic invagination stage of spermiogenesis. Because the centrioles (and the connected flagellum) do not properly interact with the nucleus or the plasma membrane, cytoplasmic invagination fails, and the flagellar compartment does not properly form. This conclusion is supported by extensive TEM data added in the revision, which is consistent with immunogold labelling and immunofluorescence data showing [axonemal] microtubules remaining and possibly degenerating in the spermatid cytoplasm. As described in their initial submission the authors also report improper assembly of the accessory structures, which based on the new data appear to be knock-on effects of earlier defects in flagellum formation. Overall, the data presented are of good quality, and the revised conclusions are more robust in light of available evidence.

Given the bewildering complexity and inherent variability of spermiogenesis, it is not surprising that some uncertainty still clouds the precise mechanisms by which CCDC183 acts. Nonetheless, the results reported in this manuscript highlight the intricate subcellular processes that need to be spatiotemporally regulated in order to transform a round spermatid into a specialized spermatozoon. This paper spotlights an overlooked step in flagellum formation and will hopefully encourage further research into the molecular mechanisms of spermiogenesis.

Comments for the author

Below are some suggestions for minor revisions:

Throughout the manuscript: It is understandable that thorough statistical analyses on TEM sections is unfeasible, but it would be very much appreciated if the authors could include N's for each observation (i.e. just state the number of sections/images each conclusion is based on). For example, in lines 160-161, the authors could add the number of observations like this: “The centriole pair connects the cell surface and nucleus in step 6 spermatids (N = X sections), but this could not be observed in Ccdc183 KO spermatids (N = Y sections)”

Abstract
Consider removing the second and third sentences, “During sperm flagellum formation, cytoplasmic invagination around the flagellum occurs, which enables flagellar compartmentalization. But little is known about mammalian cytoplasmic invagination during spermiogenesis due to the lack of suitable animal models.” The abstract reads sharper without them.

Line 35-36: “As a result, axonemal microtubules within the cytoplasm collapse during spermiogenesis”
- Revise to “As a result, the flagellar compartment does not form properly and axonemal microtubules within the cytoplasm collapse during spermiogenesis.”

© 2023. Published by The Company of Biologists under the terms of the Creative Commons Attribution License (https://creativecommons.org/licenses/by/4.0/).

Introduction
Line 47-48: The axoneme is not found in the endpiece, which only consists of singlet microtubules. Revise '[axoneme] is found throughout the tail'.

Line 50: Rewording to "Axonemal microtubules anchor several macromolecular complexes such as…" in order to emphasize that these complexes are bound to microtubules.

Line 76: This sentence is awkward, reword and shorten to: "CCDC183, also known as KIAA1984, is a testis-specific protein expressed in middle and late round spermatids."

Results
Line 104-105: "…a large number with frayed filaments…"
- Should this be "a large number of frayed filaments…"?
- Out of interest, where do the authors think these frayed microtubule filaments come from? Are they the "failed" flagella that are released from the cytoplasm of spermatids that have undergone cell death?

Line 146-148: "We then immunostained the spermatozoa with acetylated tubulin and found that microtubules were scattered in the KO cauda epididymis (Fig. 3B)."
- Figure 3B might fit better in Figure 1, as it directly follows-up from Figure 1D. The fact that the frayed filaments are microtubules can then be presented immediately after their initial description in lines 104-105.

Line 197-198: "Although it was very rare, we also observed a fibrous sheath surrounding axonal microtubules and outer dense fibers."
- axonal should be "axonemal"

Line 198-199: "However, no flagellar structure was observed around them (Fig. 4D right)."
- It is not clear what the authors mean here – is it the lack of a clear flagellar membrane?

Line 204-205: "While the connecting piece was normal, the structures of the axonemal microtubules and outer dense fibers were abnormal (Fig. 5A)"
- Do the authors have another control micrograph where the ODFs are more prominent? There may be some slight elongation from the segmented columns, but it is difficult to appreciate what the ODFs should look like at this stage with the image chosen. Also, the lack of ODFs in the KO spermatid shown in the right panel is likely related to the absence of axonemal doublets.

Line 205-206: "Furthermore, Ccdc183 KO spermatids did not exhibit correct cytoplasmic invagination around the flagellum."
- Perhaps also refer to (Fig. 5A, right, arrowheads) in the text?

Discussion
The discussion of the proposed model needs sharpening/tightening. The summary diagram in Figure 6 and the associated Figure 6 legend are straightforward and easy to understand, but the Discussion could do with some revisions for clarity.
For example, phrases such as "axonemal microtubules without a flagellum" (line 234) are confusing – it is better to explicitly state that these are the axonemal microtubules left in the cytoplasm because of failure of cytoplasmic invagination.
Likewise, the statement, "…there seem to be big differences between axonemal microtubules within the flagellum and cytoplasm" (lines 224-225) could be misleading.

Line 252-254: "One is the failure of fibrous sheath-component protein entry into the flagellum compartment, and another is a defect in protein delivery within the flagellum compartment via intra-flagellar transport (IFT)"
- Failure of cytoplasmic invagination means that the flagellar compartment does not form properly in the first place (for example there is no longer a proper transition zone). Impaired assembly of the accessory structures could be a downstream effect of this.

Materials and Methods

© 2023. Published by The Company of Biologists under the terms of the Creative Commons Attribution License (https://creativecommons.org/licenses/by/4.0/).

Could the authors add a bit more detail on how they determined what step/s of spermiogenesis each TEM image corresponds to? This would be very helpful for readers who are not in the spermiogenesis field.

Figure Legends
Figure 3
Line 651-652: reword "…moves toward the cell surface…" to "…moves toward cell surface at the opposite pole…" to clarify exactly what is going on.

Figure 6
Line 706-707: "Ccdc183 KO spermatozoon has a huge cytoplasm with abundant cytoplasmic contents including electron-dense materials (right)"
- What do such cells (spermatozoa with large cytoplasm) look like by light microscopy? The electron micrographs shown in Figure S4 are informative, but if a light microscopy image is available it might be worth adding. If this is a robust observation, it should also be added to the Discussion (around line 215-217).

Second revision

Author response to reviewers' comments

Response to reviewers
We would like to take this opportunity to thank the three expert reviewers for their constructive comments and suggestions. We wrote responses to reviewers' comments below with the original comments in black and our answers in red.

Reviewer 1 Comments for the Author:
I appreciate that the authors discuss the potential role of IFT in the new mouse model. However, the citation is not appropriate. The current citation is a review paper. There are a number of research papers about the role of IFT in mouse sperm formation, and some have been shown to be related to FS. Those original papers should be cited.
>Thank you for your comments. New sentences related to IFT have been added, and we have added original papers as references (Lanes 251-254).

Reviewer 2 Advance Summary and Potential Significance to Field:
The authors are to be congratulated on the approach they have taken to revising this manuscript. This paper was good to start with, but it is even better after the revisions. I am confident it will make a valuable contribution to the literature. The phenotype described, and thus the function of CCDC183, is very interesting and adds an important piece to the puzzle of how sperm tails are formed.

The fact that plasma membrane docking to the basal body appears to have failed yet axonemes grow (even if abnormal) is remarkable and suggestive of the cytoplasmic axoneme growth observed in drosophila - but not usually in mammals - suggesting that CCDC183 is evolutionary important. See the paper below for a summary.
https://www.ncbi.nlm.nih.gov/pmc/articles/PMC4254545/
https://pubmed.ncbi.nlm.nih.gov/26654377/

In other germ cells, as the authors have clearly shown, the axoneme is present, but the ODF and FS are absent from the ciliary lobe. I agree, that based on the current information it is not possible to determine if CCDC183 plays a role in protein entry into the ciliary compartment e.g. in the transition zone, or it plays a role in protein delivery along the axoneme/ciliary lobe e.g. via the IFT pathway. Regardless, the insights are valuable.

The discussion on the ortholog (or not) between mouse and fly CCDC183 is important.

© 2023. Published by The Company of Biologists under the terms of the Creative Commons Attribution License (https://creativecommons.org/licenses/by/4.0/).

The summary figure is helpful.

Reviewer 2 Comments for the Author:
I have no edits to suggest.

>Thank you for your valuable comments. We checked several papers related to *Drosophila* spermatids. We agree with your comment that "CCDC183 is evolutionary important". We added a new discussion in Lanes 231-236.

Reviewer 3 Advance Summary and Potential Significance to Field:
In their revised manuscript, Shimada & Ikawa present new data on CCDC183-knockout mice, re-interpreting the cellular basis of the observed male infertility. Their major new conclusion is that CCDC183 knockouts are infertile due to defects occurring around the cytoplasmic invagination stage of spermiogenesis. Because the centrioles (and the connected flagellum) do not properly interact with the nucleus or the plasma membrane, cytoplasmic invagination fails, and the flagellar compartment does not properly form. This conclusion is supported by extensive TEM data added in the revision, which is consistent with immunogold labelling and immunofluorescence data showing [axonemal] microtubules remaining and possibly degenerating in the spermatid cytoplasm. As described in their initial submission, the authors also report improper assembly of the accessory structures, which based on the new data appear to be knock-on effects of earlier defects in flagellum formation. Overall, the data presented are of good quality, and the revised conclusions are more robust in light of available evidence.

Given the bewildering complexity and inherent variability of spermiogenesis, it is not surprising that some uncertainty still clouds the precise mechanisms by which CCDC183 acts. Nonetheless, the results reported in this manuscript highlight the intricate subcellular processes that need to be spatiotemporally regulated in order to transform a round spermatid into a specialized spermatozoon. This paper spotlights an overlooked step in flagellum formation and will hopefully encourage further research into the molecular mechanisms of spermiogenesis.

Reviewer 3 Comments for the Author:
Below are some suggestions for minor revisions:
Throughout the manuscript: It is understandable that thorough statistical analyses on TEM sections is unfeasible, but it would be very much appreciated if the authors could include N's for each observation (i.e. just state the number of sections/images each conclusion is based on). For example, in lines 160- 161, the authors could add the number of observations like this: "The centriole pair connects the cell surface and nucleus in step 6 spermatids (N = X sections), but this could not be observed in Ccdc183 KO spermatids (N = Y sections)"
>We added the numbers of observed spermatids in figure legends (Figs. 3A, 3C, 3D, 4A, and 5A).

Abstract
Consider removing the second and third sentences, "During sperm flagellum formation, cytoplasmic invagination around the flagellum occurs, which enables flagellar compartmentalization. But little is known about mammalian cytoplasmic invagination during spermiogenesis due to the lack of suitable animal models." The abstract reads sharper without them.
>We removed these sentences (Line 27).

Line 35-36: "As a result, axonemal microtubules within the cytoplasm collapse during spermiogenesis"
-Revise to "As a result, the flagellar compartment does not form properly and axonemal microtubules within the cytoplasm collapse during spermiogenesis."
>We changed the above sentence as suggested (Line 33).

Introduction
Line 47-48: The axoneme is not found in the endpiece, which only consists of singlet microtubules. Revise '[axoneme] is found throughout the tail'.
>We changed it "throughout the tail except for the endpiece" (Line 46).

© 2023. Published by The Company of Biologists under the terms of the Creative Commons Attribution License (https://creativecommons.org/licenses/by/4.0/).

Line 50: Rewording to "Axonemal microtubules anchor several macromolecular complexes such as…" in order to emphasize that these complexes are bound to microtubules.
>We changed as per the reviewer's comment (Lines 47-48).

Line 76: This sentence is awkward, reword and shorten to: "CCDC183, also known as KIAA1984, is a testis-specific protein expressed in middle and late round spermatids."
>We changed as per the reviewer's comment (Lines 73-74).

Results
Line 104-105: "…a large number with frayed filaments…"-Should this be "a large number of frayed filaments…"?
-Out of interest, where do the authors think these frayed microtubule filaments come from? Are they the "failed" flagella that are released from the cytoplasm of spermatids that have undergone cell death?
>We think the frayed microtubule filaments come from dead sperm cytoplasms. The 9+2 structures are difficult to find in the epididymis by TEM analysis (Fig. S4D). However, we could find many axonemes in both cytoplasms (Fig. S5A) and compartmentalized flagellum (Fig S5B). We think the frayed microtubule filaments were released from these cytoplasms. In addition, we think normal-looking microtubules are compartmentalized flagellums without accessory structures.
We modified the above sentence as per the reviewer's comment (Line 98).
.

Line 146-148: "We then immunostained the spermatozoa with acetylated tubulin and found that microtubules were scattered in the KO cauda epididymis (Fig. 3B)."
-Figure 3B might fit better in Figure 1, as it directly follows-up from Figure 1D. The fact that the frayed filaments are microtubules can then be presented immediately after their initial description in lines 104-105.
>We agree with you on some points. However, it is difficult to predict the identity of the filament at the point of Fig. 1D. We tried to stain with tubulin because we found several collapsed microtubules (Fig. S4D). If the figures were moved, it would be difficult to achieve consistency.

Line 197-198: "Although it was very rare, we also observed a fibrous sheath surrounding axonal microtubules and outer dense fibers."
-axonal should be "axonemal"
>We modified it as per the reviewer's comment (Line 189).

Line 198-199: "However, no flagellar structure was observed around them (Fig. 4D, right)."
-It is not clear what the authors mean here – is it the lack of a clear flagellar membrane?
>We modified this sentence to make it clearer (Line 190). We want to say the structures of Fig. 4D (right) were not compartmentalized by a flagellar membrane.

Line 204-205: "While the connecting piece was normal, the structures of the axonemal microtubules and outer dense fibers were abnormal (Fig. 5A)"
-Do the authors have another control micrograph where the ODFs are more prominent? There may be some slight elongation from the segmented columns, but it is difficult to appreciate what the ODFs should look like at this stage with the image chosen. Also, the lack of ODFs in the KO spermatid shown in the right panel is likely related to the absence of axonemal doublets.
>ODF structures become more prominent as the stages progress. But KO axonemes collapse as the stages progress. Therefore, we could not find more suitable figures. Instead, we placed enlarged axoneme figures (Fig. 5A). ODF is observed as bold filaments and peripheral pairs could be observed as thin lines in the enlarged picture.

Line 205-206: "Furthermore, Ccdc183 KO spermatids did not exhibit correct cytoplasmic invagination around the flagellum."
-Perhaps also refer to (Fig. 5A, right, arrowheads) in the text?
>We added the reference (Line 197).

Discussion
The discussion of the proposed model needs sharpening/tightening. The summary diagram in Figure

© 2023. Published by The Company of Biologists under the terms of the Creative Commons Attribution License (https://creativecommons.org/licenses/by/4.0/).

6 and the associated Figure 6 legend are straightforward and easy to understand, but the Discussion could do with some revisions for clarity. For example, phrases such as "axonemal microtubules without a flagellum" (line 234) are confusing - it is better to explicitly state that these are the axonemal microtubules left in the cytoplasm because of failure of cytoplasmic invagination. Likewise, the statement, "…there seem to be big differences between axonemal microtubules within the flagellum and cytoplasm" (lines 224-225) could be misleading.
>Thank you for your comment. We use "compartmentalized axoneme" and "cytosol-exposed axoneme" instead of "axoneme within the flagellum" and "axoneme within the cytoplasm" (Lines 214-216). In addition, we modified Lines 227-231 for clarity.

Line 252-254: "One is the failure of fibrous sheath-component protein entry into the flagellum compartment, and another is a defect in protein delivery within the flagellum compartment via intra- flagellar transport (IFT)"
-Failure of cytoplasmic invagination means that the flagellar compartment does not form properly in the first place (for example there is no longer a proper transition zone). Impaired assembly of the accessory structures could be a downstream effect of this.
>We agree with your opinion. We modified the sentences (Lines 247-250).

Materials and Methods
Could the authors add a bit more detail on how they determined what step/s of spermiogenesis each TEM image corresponds to? This would be very helpful for readers who are not in the spermiogenesis field.
>We added the evaluation method to the manuscript (Lines 410-413).

Figure Legends
Figure 3
Line 651-652: reword "…moves toward the cell surface…" to "…moves toward cell surface at the opposite pole…" to clarify exactly what is going on.
>We modified it as per the reviewer's comment (Line 656).

Figure 6
Line 706-707: "Ccdc183 KO spermatozoon has a huge cytoplasm with abundant cytoplasmic contents including electron-dense materials (right)"
-What do such cells (spermatozoa with large cytoplasm) look like by light microscopy? The electron micrographs shown in Figure S4 are informative, but if a light microscopy image is available, it might be worth adding. If this is a robust observation, it should also be added to the Discussion (around line 215-217).
>This is a very important point. We checked the testicular sections in KO and found abnormal cytoplasm in the posterior side of elongated spermatids (Fig. 2A). We added this information in Lines 116-118.

Third decision letter

MS ID#: DEVELOP/2023/201724

MS TITLE: CCDC183 is essential for cytoplasmic invagination around the flagellum during spermiogenesis and male fertility

AUTHORS: Keisuke Shimada and Masahito Ikawa

ARTICLE TYPE: Research Article

I am happy to tell you that your manuscript has been accepted for publication in Development, pending our standard ethics checks.

© 2023. Published by The Company of Biologists under the terms of the Creative Commons Attribution License (https://creativecommons.org/licenses/by/4.0/).
